# Nurse-led intervention for improving quality of life of breast cancer patients: systematic review and meta-analysis

**DOI:** 10.1186/s12912-026-04505-2

**Published:** 2026-05-14

**Authors:** Jun Fu, Xinqing Zhu, Jiali Feng

**Affiliations:** 1 Department of Emergency Medicine, Shandong Provincial Third Hospital, Shandong University, No. 11 Wuyingshan Middle Road, Tianqiao District, Jinan City, Shandong Province 250031 P. R. China; 2 Department of Thyroid and Breast Surgery, Shandong Provincial Third Hospital, Shandong University, No. 11 Wuyingshan Middle Road, Tianqiao District, Jinan City, Shandong Province 250031 P. R. China

**Keywords:** Meta-analysis, Nurse, Quality of life

## Abstract

**Background:**

Breast cancer treatment often impacts patients’ quality of life (QoL), with symptoms ranging from fatigue and pain to emotional and functional disturbances. Nurse-led interventions have been proposed to mitigate these effects, yet comprehensive evidence of their efficacy remains varied. Hence, this meta-analysis evaluated the effectiveness of nurse-led interventions on various QoL domains among breast cancer patients.

**Methods:**

Search strategy included databases such as PubMed, Scopus, Cochrane Library, CINAHL, and Google Scholar. Random-effects meta-analyses were performed to estimate standardized mean difference (SMD); heterogeneity was quantified using I² and τ² with 95% confidence intervals, and small-study effects were explored using funnel plots and Egger’s test. Study quality/risk of bias was appraised with RoB 2 for randomized trials. All analyses were conducted in Stata (version 14.2, StataCorp).

**Results:**

Interventions showed modest, non-significant improvement in global health status (SMD = 0.265; 95%CI: -0.019 to 0.549) and functional status (SMD = 0.241; 95%CI: -0.009 to 0.491). Significant reduction was noted in fatigue (SMD=-1.328; 95%CI: -2.550 to -0.107). Other outcomes like symptom scores, role functioning, emotional functioning, pain, dyspnea, and nausea/vomiting demonstrated non-significant trends towards improvement. High heterogeneity was observed across most outcomes, indicating variability in intervention effects.

**Conclusion:**

Nurse-led interventions may offer marginal improvements in certain QoL domains for breast cancer patients, particularly in reducing fatigue. The variability in efficacy across different outcomes suggests need for personalized approaches and further research to optimize these interventions for broader application in oncology care settings.

**Clinical trial number:**

Not applicable.

**Supplementary Information:**

The online version contains supplementary material available at 10.1186/s12912-026-04505-2.

## Summary statement

**What is already known about this topic?:**

Nurse-led interventions have been suggested to improve the quality of life (QoL) in breast cancer patients by addressing physical, emotional, and psychosocial challenges.

Previous studies indicate that these interventions may offer benefits, but results have been inconsistent, with varying effects on different QoL domains.

There is a lack of comprehensive evidence synthesizing the impact of nurse-led interventions on specific QoL outcomes, highlighting a knowledge gap.

**What this paper adds:**

This meta-analysis provides a detailed evaluation of the effectiveness of nurse-led interventions on multiple QoL domains for breast cancer patients.

Significant reduction in fatigue was observed, showcasing a specific benefit of nurse-led care.

The study highlights modest improvements in global health status and functional status, though these were not statistically significant, and identifies substantial variability in intervention effects.

**Supplementary Information:**

The online version contains supplementary material available at 10.1186/s12912-026-04505-2.

## The implications of this paper

How can nurse-led interventions be integrated into comprehensive cancer care plans to improve QoL for breast cancer patients?

By including nurse-led interventions as part of standard oncology care, particularly for managing symptoms like fatigue and pain, healthcare providers can potentially enhance patient well-being.

What elements of nurse-led interventions are most effective in improving patient outcomes?

The study suggests that personalized, continuous support and education are critical components, warranting further research to standardize and optimize these interventions.

How should future research be directed to better understand and implement nurse-led interventions in oncology settings?

Future studies should focus on larger, well-designed RCTs, consistent outcome measures, and long-term impacts, as well as exploring patient-specific characteristics and cost-effectiveness of interventions.

**Supplementary Information:**

The online version contains supplementary material available at 10.1186/s12912-026-04505-2.

## Introduction

Breast cancer remains a major public-health burden: in 2022 an estimated 2.3 million women were newly diagnosed and ~ 670,000 died globally, making it the most diagnosed cancer in women and a leading cause of cancer death [5]. Five-year global prevalence now exceeds 8 million persons living with breast cancer. In India, ~ 192,000 new cases and ~ 98,000 deaths were estimated in 2022, with a five-year prevalence of ~ 526,000, underscoring the need for scalable, effective survivorship and symptom-management strategies. Breast cancer, not only poses significant health risks but also profoundly impacts the quality of life of those diagnosed [32]. The journey from diagnosis through treatment and beyond is fraught with challenges that extend beyond physical health, encompassing emotional, psychological, and social dimensions [9]. The quality of life (QoL) for breast cancer patients is influenced by various factors, including pain, fatigue, emotional distress, and the side effects of treatment, which can persist long after the completion of medical interventions [22]. This complexity necessitates a comprehensive approach to care, one that addresses the multifaceted needs of patients to improve their overall well-being [20].

Within this context, nurse-led interventions have gained recognition for their potential to enhance the quality of life of breast cancer patients [10]. Nurses play a pivotal role in oncological care teams, providing continuous support that spans the entirety of the patient care spectrum from diagnosis to treatment and follow-up care [19]. Their unique position allows them to deliver interventions that are not only medically supportive but also tailored to address the emotional and psychosocial aspects of cancer care [26].

Nurse-led interventions often encompass a range of activities, including patient education, psychological support, symptom management, and the facilitation of communication between patients and their healthcare providers [15]. These interventions are designed to empower patients, enhance their coping strategies, and provide them with the tools needed to manage the physical and emotional challenges posed by breast cancer [4]. The goal is to foster a better quality of life by mitigating the distressing symptoms and side effects associated with breast cancer and its treatment [29].

The effectiveness of these interventions, however, can vary widely [1, 2, 31]. Differences in intervention design, the specific training and competencies of the nursing staff, and the individual needs and backgrounds of patients can all influence outcomes. Given the personal and societal burdens associated with breast cancer, there is a pressing need to understand which aspects of nurse-led interventions are most effective in improving patient-reported outcomes, particularly quality of life [1, 2, 31].

This variability in intervention effectiveness points to the necessity for a review that examines the breadth of research on nurse-led interventions aimed at improving the quality of life for breast cancer patients. Such an analysis can provide a comprehensive overview of the evidence, highlight the most effective practices, and identify gaps in current research. By evaluating the outcomes of various studies, the key components of successful interventions can be identified and offer guidance for their implementation in clinical practice.

Ultimately, there is a need to enhance the body of knowledge regarding the impact of nurse-led interventions on the quality of life of breast cancer patients. There is a need to ascertain whether these interventions can consistently improve patient outcomes and which elements are most beneficial. This insight is crucial for developing standardized practices that can be implemented across diverse healthcare settings to ensure that all breast cancer patients receive the support necessary to navigate their treatment journey with enhanced comfort and dignity.

By focusing on nurse-led interventions specifically tailored to improving the quality of life for breast cancer patients, this study addresses a vital component of cancer care. The findings of this meta-analysis could significantly influence how nursing care is structured in oncology, potentially leading to improved patient outcomes and more effective utilization of healthcare resources. In doing so, it contributes to the broader goal of making cancer care more patient-centered and holistic, ultimately enhancing the quality of life for those affected by breast cancer. Hence, this review was done to determine the effectiveness of nurse-led interventions for improving quality of life of the breast cancer patients.

## Methods

This review was done in accordance with PRISMA guidelines to maintain a high standard of review integrity and allow for reproducibility [21].

### Design

Systematic review and meta-analysis of randomized controlled trials.

### Eligibility criteria

Studies satisfying the following set of criteria were eligible for inclusion: Studies involving breast cancer patients aged ≥ 18 years, undergoing any stage of treatment or post-treatment follow-up.; Studies examining the impact of nurse-led interventions aimed at improving quality of life compared to usual care or no intervention;

We operationalised nurse-led interventions as structured, health-service or behavioural programmes for which: (i) the lead provider is a nurse (registered nurse, clinical nurse specialist, nurse practitioner, breast care nurse, or nurse navigator) who holds primary responsibility for intervention delivery/titration; (ii) ≥ 50% of planned patient-facing contacts are delivered by nurses (in person, telephone, or telehealth); and (iii) content extends beyond usual nursing care to include one or more prespecified components such as symptom self-management coaching, psychoeducation/counselling, survivorship planning, case management/patient navigation, adherence support, relaxation/massage or supportive touch, nurse-supervised exercise/rehabilitation, or structured tele-follow-up. We included team-based models when the nurse was the designated coordinator/lead with an algorithm or protocol defining actions and escalation. We excluded programmes primarily led by non-nurse professionals (e.g., physician-led, psychologist-led, physiotherapist-led, dietitian-led) without nurse leadership; lay-health-worker or volunteer-only interventions; usual care or unstructured discharge advice; purely administrative reminder calls; studies where the nurse’s role was limited to data collection; and interventions where < 50% of direct contact time was nurse-delivered. Pharmacologic, surgical, radiotherapy, or device-based interventions without a defined nurse-led component were also excluded.

Primary outcomes focused on measures of quality of life, including global health/overall quality of life, functional status, symptom score, role functioning, emotional functioning, fatigue, pain, dyspnea, nausea and vomiting, as reported through validated QoL instruments like the EORTC QLQ-C30; Included study designs were randomized controlled trials (RCTs) that provided data on the effectiveness of nurse-led interventions.

### Information sources

PubMed, Scopus, Cochrane Library, CINAHL, and Google Scholar.

### Search strategy

Search terms included “Breast Cancer,” “Nurse-Led Care,” “Quality of Life,” “Patient Support,” and “Survivorship Care.” No restrictions were imposed on research selection based on ethnicity, race, place of residence, or sample size. No language restrictions were applied, and the literature search was comprehensive, spanning from the inception of each database (January 1956) till December 2024. Full search strategy is provided in Supplementary Appendix.

### Study screening process

All records from the searches conducted in PubMed, Scopus, Cochrane Library, CINAHL, and Google Scholar were first exported to EndNote software and subsequently downloaded to a dedicated, secure folder on our institutional server. This ensured that all retrieved titles and abstracts were stored systematically for further review.

Duplicate records were automatically removed using EndNote’s deduplication features. Following duplicate removal, two independent reviewers conducted a title and abstract screening. In this phase, each record was evaluated against pre-specified inclusion and exclusion criteria. Records meeting all the criteria were selected for full-text retrieval and further appraisal. Any disagreements were resolved through a consensus meeting which included a senior oncology nurse specialist. The study selection followed PRISMA guidelines to maintain a high standard of review integrity and allow for reproducibility [21].

### Data extraction

Two reviewers independently extracted data in duplicate using a pre-piloted, standardized form; disagreements were resolved by consensus or, when needed, a third reviewer. The form was developed a priori from the PICOS framework and relevant reporting checklists (CONSORT), then refined after a calibration exercise on a subset of studies to capture study characteristics including sample size of intervention and control group, participant criteria, intervention/comparator details, outcome measures/time points, effect estimates, and risk-of-bias items. Data was extraction from each included study into Microsoft Excel program. Participant demographics (age, cancer stage, treatment phase) and details about the nurse-led interventions (nature of the intervention, frequency, and overall duration) were recorded. Outcome measures such as quality of life indices in both intervention and control group were carefully documented.

### Risk of bias assessment

The risk of bias assessment of the studies was carried out by two evaluators utilizing two distinct bias assessment tools. For RCTs, the Cochrane Collaboration’s Risk of Bias 2 (RoB-2) tool [28] was used, assessing potential biases in areas such as randomization processes, deviations from intended interventions, management of missing outcome data, outcome measurement, and result reporting. Studies were subsequently categorized as having ‘low,’ ‘high,’ or ‘some concerns’ regarding their risk of bias, ensuring a comprehensive and meticulous quality review of the collected evidence.

### Statistical analysis

The statistical analysis for this study was conducted using STATA, version 14.2 (StataCorp, College Station, TX). Given that all outcomes were continuous, the analysis involved calculating the pooled weighted mean difference (WMD) or standardized mean difference (SMD) along with 95% confidence interval (CI), based on mean and standard deviation (SD) in both intervention and control groups. The quality-of-life, functional and emotional outcomes in the included studies were measured using a range of validated instruments. Given this heterogeneity in measurement tools, we employed SMD to synthesize the results. SMD was calculated by dividing the difference in means between the intervention and control groups by the pooled standard deviation. This method standardizes the effect sizes across studies regardless of the scale used, allowing us to combine disparate outcome values into a single metric. All instruments included in the review were selected based on their established validity and reliability in assessing quality-of-life in breast cancer populations. Thus, the use of SMD not only facilitated the combination of results from different measurement scales but also ensured that the summary estimates were robust and comparable.

Random-effects model was employed, using the inverse variance method to manage variations among the included studies [8]. To assess heterogeneity, or the differences in results across studies, various methods were utilized including visual examination of forest plots to check for overlaps in CIs, chi-square tests, and the I^2^ statistic, which measures the proportion of total variation attributable to between-study differences. The classification of heterogeneity was done based on value of I-squared statistic: 0%-25% as low, 25%-50% as medium and 51%-75% as high and above 75% as substantial heterogeneity. For evaluating publication bias, a variety of methods were used. Egger’s test was applied to check for asymmetry in the data, which may suggest the presence of bias. Funnel plots served as a visual tool to evaluate bias by plotting the effect sizes of the studies against their precision [8].

#### Interpretation of effect size and clinical importance

Because multiple QoL instruments were used across trials, we pooled standardized mean differences (SMD; Hedges g) and interpreted magnitude using conventional thresholds (trivial < 0.20; small 0.20–<0.50; moderate 0.50–<0.80; large ≥ 0.80). To operationalise clinical importance, we applied instrument-specific minimal clinically important differences (MCIDs) when available (e.g., EORTC QLQ-C30 global health/QoL: ~5–10 points for a small but important difference; FACT-B total score: ~7–8 points; HADS subscales: ~1.5–1.7 points; EQ-5D index: ~0.07–0.10; SF-36 domains: ~3–5 points). For outcomes reported on multiple scales and meta-analysed as SMDs, we used two complementary strategies: (1) a distribution-based threshold of 0.30 SD as a conservative MCID proxy when anchor-based MCIDs were unavailable; and (2) where ≥ 40% of trials within an analysis used the same instrument, we back-transformed the pooled SMD into that instrument’s units using the pooled baseline SD from those trials and compared the resulting mean difference to the published MCID. We considered effects “clinically important” when point estimates met or exceeded the relevant MCID and “marginal” when statistically significant effects did not reach the MCID. Alongside I², we report τ² and 95% prediction intervals to contextualise between-study heterogeneity and the expected range of true effects in new settings.

## Results

### Search results

Database searches yielded 2,576 records (PubMed *n* = 892; Scopus *n* = 832; CINAHL *n* = 212; Cochrane *n* = 119; Google Scholar *n* = 521). After removing 722 duplicates (no automation tools used), 1,854 unique titles/abstracts were screened, of which 1,771 were excluded as clearly irrelevant to the review question. We retrieved 83 full-text reports and assessed them for eligibility; none were unretrieved. Of these, 68 reports were excluded for prespecified reasons: different intervention (not nurse-led or not meeting our operational criteria; *n* = 47), different outcome (did not report the predefined QoL/symptom endpoints; *n* = 19), and data for analysis not available (insufficient extractable data; *n* = 2). Ultimately, 15 studies met the inclusion criteria for the review (Fig. 1A) ([1, 2, 14, 16–18, 23–25, 27, 30, 31, 33]).

**Fig. 1A:**
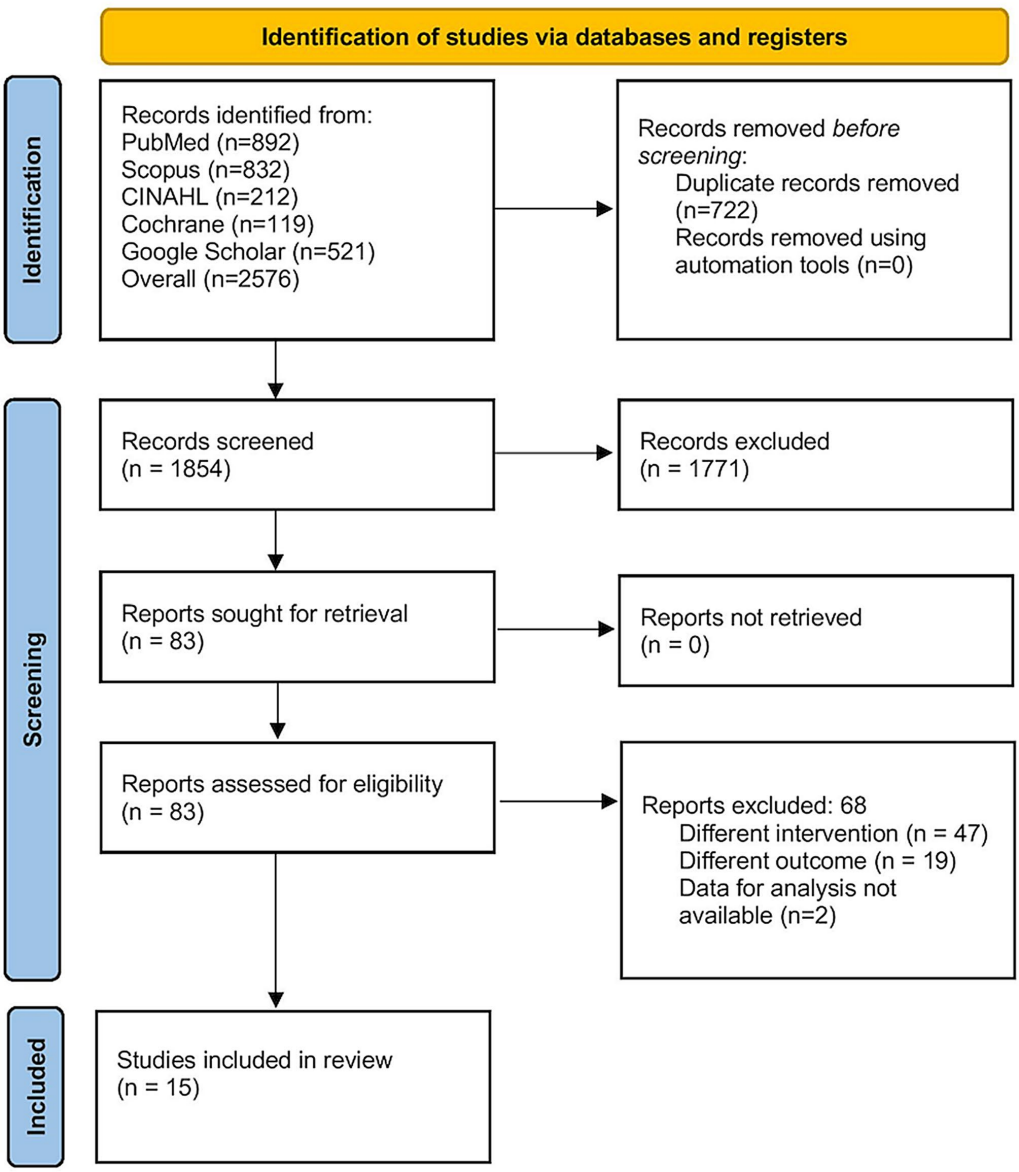
PRISMA flowchart

**Fig. 1B:**
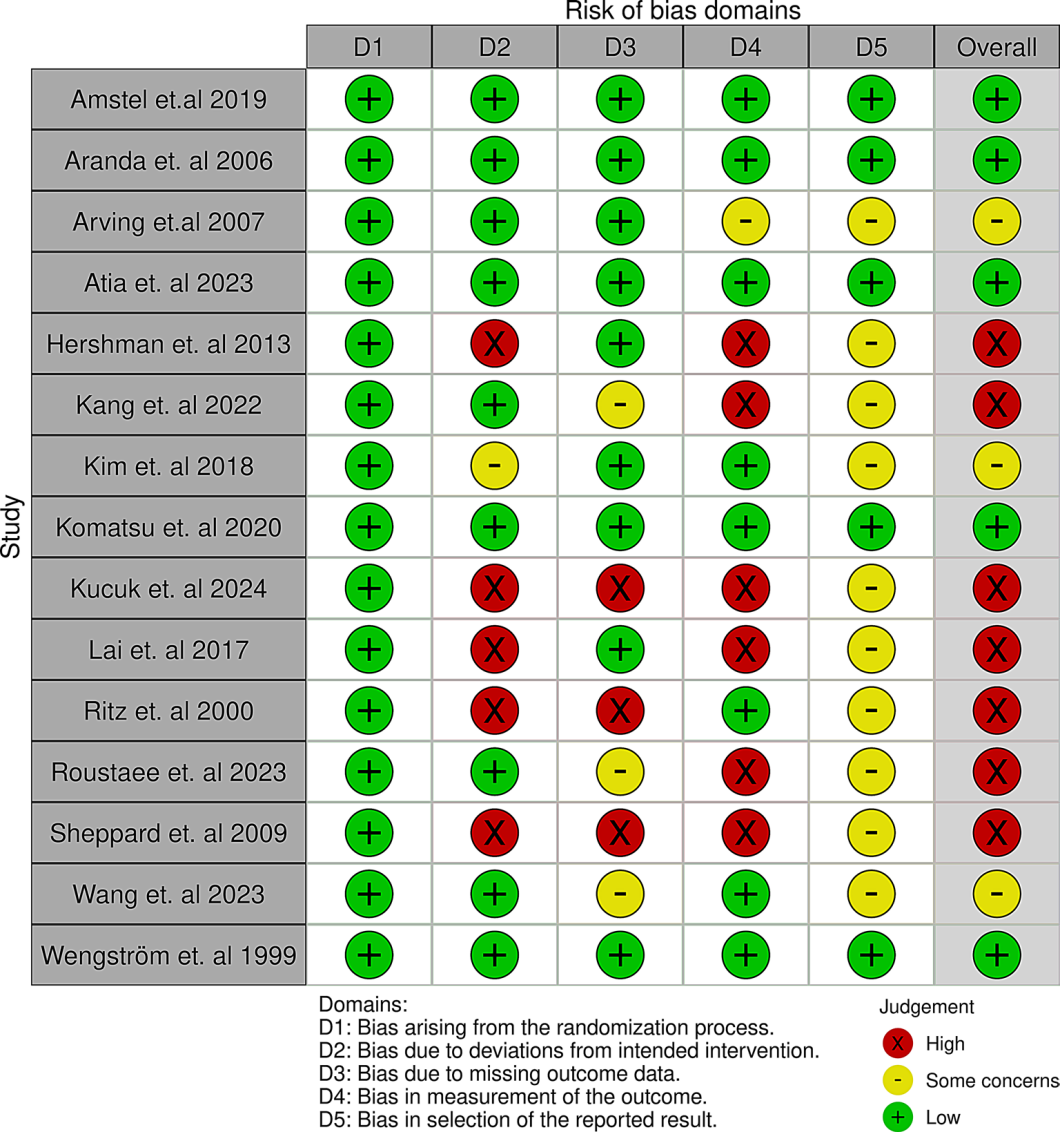
Risk of bias assessment based on Cochrane Risk of bias-2 (RoB-2)

### Characteristics of the included studies

Table 1 reports the characteristics of the included studies. In total, 15 RCTs were included, representing a diverse international sample conducted in Australia, Sweden, Egypt, USA, Korea, South Korea, Japan, Turkey, China, Netherlands, Iran, and UK. Sample sizes ranged from 52 to 561 participants. The nurse-led interventions varied considerably and included psychosocial nursing interventions, individualized psychosocial support, exercise rehabilitation programs, psychological intervention programs, medication self-management programs, and mirror therapy. Quality of life was assessed using a range of validated instruments such as different versions of the EORTC QLQ (e.g., QLQ-C30, QLQ-BR23), Functional Assessment of Cancer Therapy (FACT) series, and other measures including the Profile of Mood States (POMS) and various fatigue and stress scales. Reported mean ages of study participants generally fell in the mid-40s to mid-50s, although one study included older patients (with mean ages up to 71 years). In terms of methodological quality, five studies were rated as having a low risk of bias, while three studies were identified with some concerns and seven studies with high risk of bias (Fig. 1B).

**Table 1:** Characteristics of the included studies

Study identifier	Country	Study design	Sample size	Study participants	Mean age (in years)	Intervention details	Comparator details	Outcome scale assessed
van Ploos et al. [23]	Netherlands	RCT	194	Women who were age ≥ 18 years with histology-proven invasive breast cancer and eligible for curative treatment.	52.5 (30–86) / 53.1 (26–75)	Regular nurse-led distress screening and discussion	Comparator group patients consists of patients receiving usual care	EORTC QLQ C30
Aranda et al. [1]	Australia	RCT	105	Women with breast cancer That was newly diagnosed at an advanced stage	57 (34–85) / 55 (36 − 82)	Psychosocial nursing interventions	Standard Care consisted of referral of patients	EORTC QLQ-30 version 2
Arving et al. [2]	Sweden	RCT	179	Patients with breast cancer, living in Uppsala County and about to start adjuvant treatment at the Department of Oncology.	55 (34–72) 55 (23–75) 55 (25–87)	Interventions during survivorship: Individual Psychosocial Support	Standard care included regular contact with the patient’s oncologist and medical staff.	EORTC QLQ-C30;
Atia et al. [3]	Egypt	RCT	60	Adult breast cancer patients who have completed their initial surgical and oncological treatments.	42.63 ± 9.07 44.53 ± 5.03	Nursing intervention based on acceptance commitment therapy on stress, marital adjustment, sleep quality, and fatigue among patients with breast cancer.	Control group consists of patients who receive usual care and did not receive any therapy	Marital adjustment Scale
Hershman et al. [11]	USA	RCT	317	Women who had a history of stage 0–III breast cancer and Were within 6 weeks of completion of initial adjuvant Treatment	54.9 (10.9)/ 53.7 (12.1)	Clinic-based survivorship Intervention	Control group consists of patients who receive usual care	Physical health awareness scale
Kang et al. [14]	Korea	RCT	72	Women with a confirmed diagnosis of breast cancer according to American Joint Committee On Cancer	46.9 (8.3)/ 48.1 (7.9)	4-Week Nurse-Led Exercise Rehabilitation Program	Control group consists of patients who receive physical therapist-supervised program	EORTC QLQ-C30
Kim et al. [16]	South Korea	RCT	152	Patients had been diagnosed with breast cancer 22–23 days prior to the study, on average, and more than half had stage II cancer during the study period.	48	Nurse-led psychological intervention program	Control group consists of patients who receive usual care	Korean version of Profile of EORTC QLQ-C30
Komatsu et al. [17]	Japan	RCT	164	Patients with metastatic breast cancer who had been newly prescribed an oral chemotherapy or a targeted Therapy agent.	7.14(8.31)/ 9.71(9.36)	Nurse-led medication self-management programme	Conventional care, including explanations of and instructions on oral chemotherapy and information on treatment-related toxicity.	General Self-Efficacy Scale Japanese version of the 36-item Functional Assessment of Cancer Therapy-Breast, the Japanese version of the 6-item Kessler 6
Kucuk et al. [33]	Turkey	RCT	86	Women who were older than 18years, newly diagnosed with BC and had stage II or III BC.	47.14 (8.31) / 49.71 (9.36)	Nurse-Led Supportive Care Program	Control group received only routine treatment	EORTC-QLQ-C30.
Lai et al. [18]	China	RCT	399	Women who were at least 18years old, having a diagnosis of primary breast cancer, receiving Adjuvant chemotherapy for the first time, Karnofsky Performance Scale score of at least 60.	T = 51.13 (9.02) I = 51.55 (8.82) C = 50.58 (9.25)	Nurse-Led Care Program for Breast Cancer Patients in a Chemotherapy	Control group received only routine hospital care	Functional Assessment of Cancer Therapy General
Ritz et al. [24]	USA	RCT	561	Female breast cancer patients > 18 years old who were newly diagnosed	55.7 / 55.3	Advanced nursing care which consists of follow-up care and Interventions based on brooten’s work and the standards of advanced practice in oncology nursing	Control group received only standard medical care	Functional Assessment of Cancer Therapy
Roustaee et al. [25]	Iran	RCT	90	Women with unilateral Mastectomy who referred to the only breast cancer clinic in Shiraz.	49.8 (9.89) 48.23 (8.8)	Mirror therapy effect on shoulder pain and Disability and quality of life	Control group received only sham therapy	EORTC-QOL QLQ-BR23 scale
Sheppard et al. [27]	UK	RCT	495	Patients diagnosed 2 years prior, who were not undergoing current treatment (except endocrine therapy), with no clinical signs of recurrence	71	Breast cancer follow up:6-monthly reviews received further Follow up appointments for clinical review recurring every 6 months with an annual mammogram.	Control group received routine hospital based 6-monthly clinical review	Functional Assessment of Cancer Therapy questionnaire with the addition of the breast and endocrine subscales
Wang et al. [30]	China	RCT	52	Patients who had been first diagnosed breast cancer and subsequently underwent mastectomy.	53.1 (48.0_61.0)/ 56.2(48.3_62.0)	Upper Limb Rehabilitation Program on Quality of Life	Control group received only standard medical care	EORTC-QOL QLQ-BR23 scale
Wengström et al. [31]	Sweden	RCT	134	Breast cancer patients receiving radiation therapy	58 (39–76)/ 61 (37–83)	Nursing intervention on subjective Distress, side effects and quality of life	Control group received only standard care	Cancer Rehabilitation Evaluation System

EORTC QLQ BR23 – European Organisation for Research and Treatment of Cancer Quality of Life Questionnaire – Breast Cancer Module (23 items); QOL – Quality of Life; RCT – Randomized controlled trial; UK – United Kingdom; USA – United States of America

### Global health status/overall quality of life

**Fig. 2:**
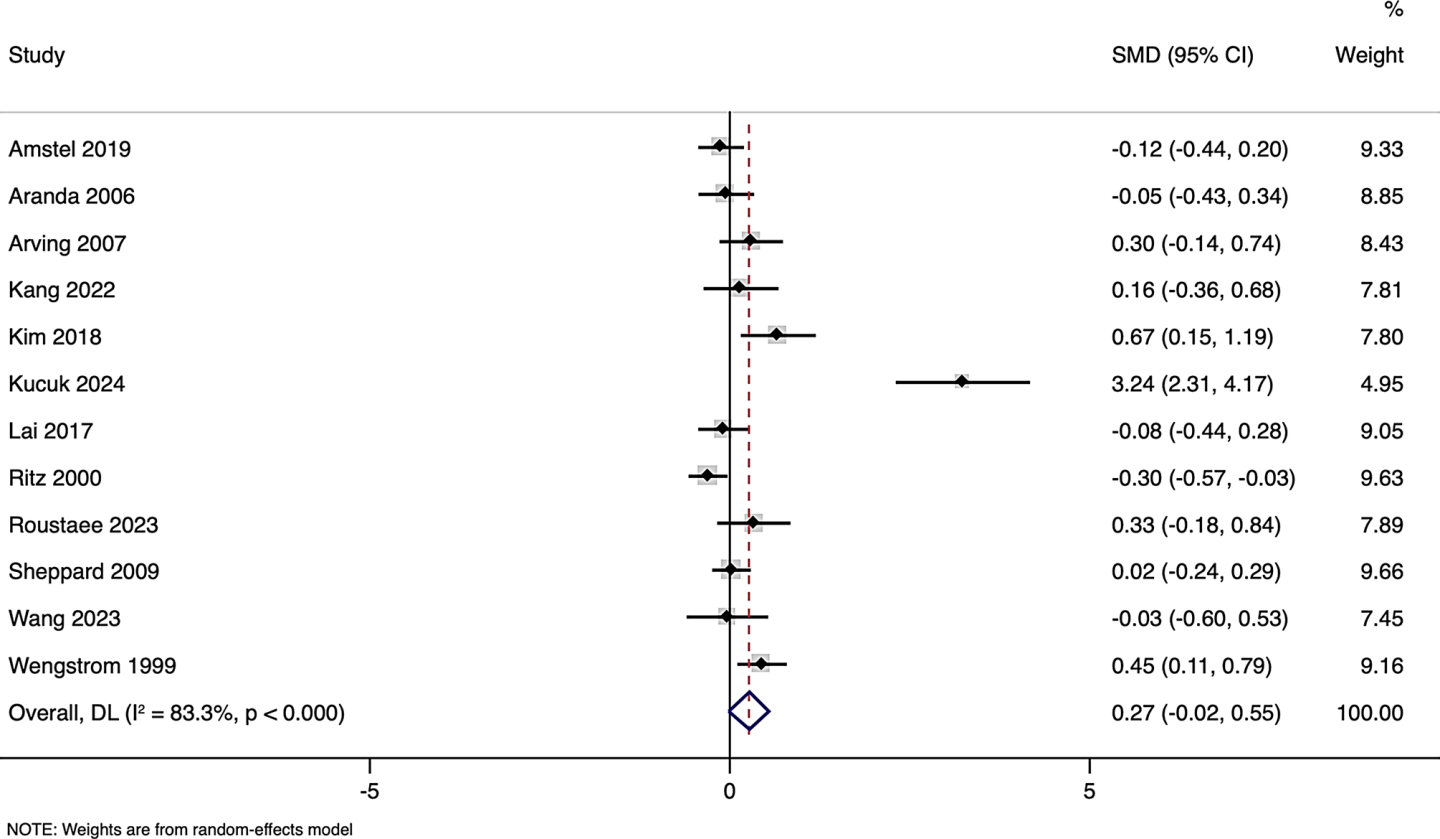
Forest plot showing the effectiveness of nurse-led interventions for global health status/overall quality of life amongst breast cancer patients. *Footnotes*: DL, DerSimonian–Laird random‑effects model; SMD, standardized mean difference; CI, confidence interval; %, inverse‑variance weight of each study. Squares represent study‑specific effect estimates (SMD) and their size is proportional to the study weight; Horizontal lines indicate the 95% confidence intervals; The solid vertical line denotes the line of no effect (SMD = 0); The dashed vertical line indicates the pooled effect estimate; Diamonds represent the overall pooled effect size with their width corresponding to the 95% confidence interval; Black markers and lines show individual studies; the blue diamond shows the pooled random‑effects estimate

This meta-analysis examined the impact of nurse led interventions on global health status/overall quality of life amongst breast cancer patients across 12 studies with 1,283 participants, reporting a pooled SMD of 0.265 (95% CI: -0.019 to 0.549) (Fig. 2). Despite a non-significant overall effect (*p* = 0.068), substantial heterogeneity was observed (I² = 83.3%), suggesting variability in intervention outcomes across studies. Funnel plot was asymmetrical (Supplementary Fig. 1) and it was further confirmed by Egger’s test (*p* = 0.006). Although point estimates favoured nurse-led interventions, the pooled SMD corresponded to a change below the lower bound of the published MCID for the dominant QoL instrument in this analysis, supporting our description of the overall effect as clinically marginal despite statistical significance.

### Functional status

**Fig. 3:**
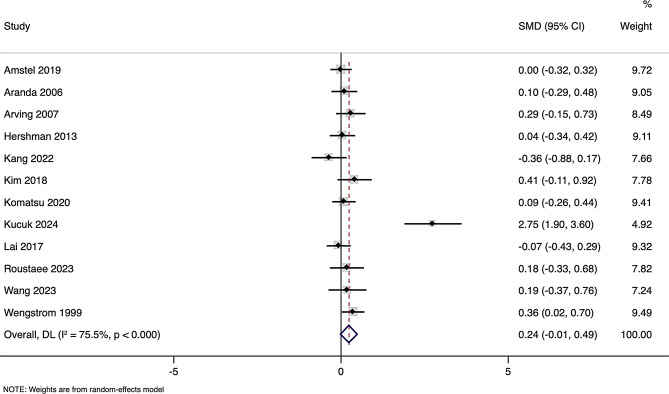
Forest plot showing the effectiveness of nurse-led interventions for functional status amongst breast cancer patients. *Footnotes*: DL, DerSimonian–Laird random‑effects model; SMD, standardized mean difference; CI, confidence interval; %, inverse‑variance weight of each study. Squares represent study‑specific effect estimates (SMD) and their size is proportional to the study weight; Horizontal lines indicate the 95% confidence intervals; The solid vertical line denotes the line of no effect (SMD = 0); The dashed vertical line indicates the pooled effect estimate; Diamonds represent the overall pooled effect size with their width corresponding to the 95% confidence interval; Black markers and lines show individual studies; the blue diamond shows the pooled random‑effects estimate

This meta-analysis assessed the effect of nurse-led interventions on the functional status of breast cancer patients, incorporating data from 12 studies with 1,092 participants. The analysis revealed a pooled SMD of 0.241 (95% CI: -0.009 to 0.491), suggesting a modest improvement in functional status, although this result was not statistically significant (*p* = 0.059) (Fig. 3). The studies exhibited considerable heterogeneity (I² = 75.5%), indicating variability in how interventions impacted functional outcomes across different settings. Funnel plot was asymmetrical (Supplementary Fig. 2) and it was further confirmed by Egger’s test (*p* = 0.035).

### Symptom score

**Fig. 4:**
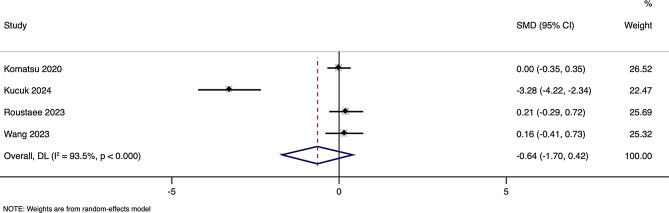
Forest plot showing the effectiveness of nurse-led interventions for symptom score amongst breast cancer patients. *Footnotes*: DL, DerSimonian–Laird random‑effects model; SMD, standardized mean difference; CI, confidence interval; %, inverse‑variance weight of each study. Squares represent study‑specific effect estimates (SMD) and their size is proportional to the study weight; Horizontal lines indicate the 95% confidence intervals; The solid vertical line denotes the line of no effect (SMD = 0); The dashed vertical line indicates the pooled effect estimate; Diamonds represent the overall pooled effect size with their width corresponding to the 95% confidence interval; Black markers and lines show individual studies; the blue diamond shows the pooled random‑effects estimate

This meta-analysis assessed the impact of nurse-led interventions on symptom scores among breast cancer patients, involving four studies with a total of 276 participants. The pooled SMD was − 0.641 (95% CI: -1.698 to 0.415), indicating a non-significant reduction in symptom scores (*p* = 0.234) (Fig. 4). The analysis demonstrated substantial heterogeneity (I² = 93.5%), reflecting significant variability in the intervention effects across studies. Publication bias was not assessed due to limitation in the number of studies.

### Role functioning

**Fig. 5:**
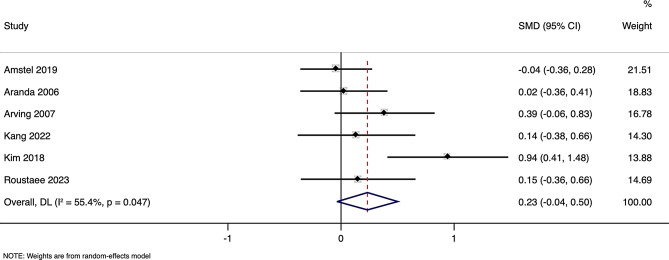
Forest plot showing the effectiveness of nurse-led interventions for role functioning amongst breast cancer patients. *Footnotes*: DL, DerSimonian–Laird random‑effects model; SMD, standardized mean difference; CI, confidence interval; %, inverse‑variance weight of each study. Squares represent study‑specific effect estimates (SMD) and their size is proportional to the study weight; Horizontal lines indicate the 95% confidence intervals; The solid vertical line denotes the line of no effect (SMD = 0); The dashed vertical line indicates the pooled effect estimate; Diamonds represent the overall pooled effect size with their width corresponding to the 95% confidence interval; Black markers and lines show individual studies; the blue diamond shows the pooled random‑effects estimate

This meta-analysis evaluated the impact of nurse led interventions on role functioning among 515 breast cancer patients across six studies. The pooled SMD was 0.233 (95% CI: -0.036 to 0.502), suggesting a non-significant improvement in role functioning (*p* = 0.089) (Fig. 5). Moderate heterogeneity was observed (I² = 55.4%), indicating some variation in effects across studies. This variability may reflect differences in intervention types, populations studied, or other study-specific characteristics. Publication bias was not assessed due to limitation in the number of studies.

### Emotional functioning

**Fig. 6:**
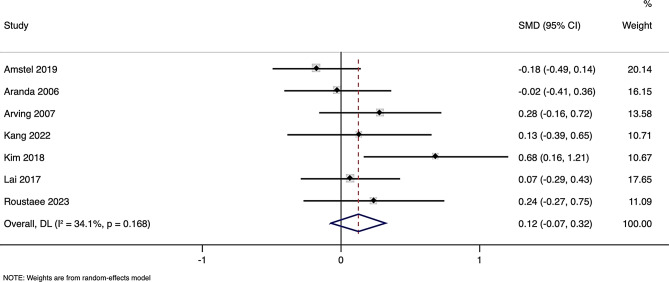
Forest plot showing the effectiveness of nurse-led interventions for emotional functioning amongst breast cancer patients. *Footnotes*: DL, DerSimonian–Laird random‑effects model; SMD, standardized mean difference; CI, confidence interval; %, inverse‑variance weight of each study. Squares represent study‑specific effect estimates (SMD) and their size is proportional to the study weight; Horizontal lines indicate the 95% confidence intervals; The solid vertical line denotes the line of no effect (SMD = 0); The dashed vertical line indicates the pooled effect estimate; Diamonds represent the overall pooled effect size with their width corresponding to the 95% confidence interval; Black markers and lines show individual studies; the blue diamond shows the pooled random‑effects estimate

This meta-analysis assessed the impact of nurse-led interventions on emotional functioning among 635 participants across seven studies. The pooled SMD was 0.125 (95% CI: -0.072 to 0.321), indicating a non-significant enhancement in emotional functioning (*p* = 0.214) (Fig. 6). The analysis demonstrated low to moderate heterogeneity (I² = 34.1%), suggesting a relatively consistent effect across the included studies. Publication bias was not assessed due to limitation in the number of studies.

### Fatigue

**Fig. 7:**
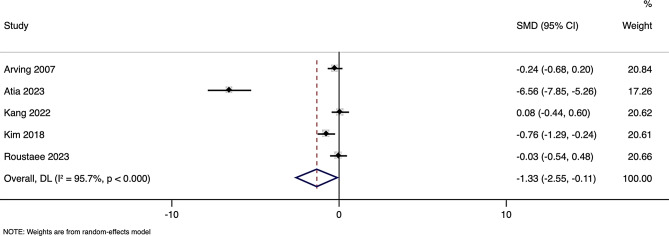
Forest plot showing the effectiveness of nurse-led interventions for fatigue amongst breast cancer patients. *Footnotes*: DL, DerSimonian–Laird random‑effects model; SMD, standardized mean difference; CI, confidence interval; %, inverse‑variance weight of each study. Squares represent study‑specific effect estimates (SMD) and their size is proportional to the study weight; Horizontal lines indicate the 95% confidence intervals; The solid vertical line denotes the line of no effect (SMD = 0); The dashed vertical line indicates the pooled effect estimate; Diamonds represent the overall pooled effect size with their width corresponding to the 95% confidence interval; Black markers and lines show individual studies; the blue diamond shows the pooled random‑effects estimate

This meta-analysis examined the impact of nurse-led interventions on fatigue among 317 participants across five studies. The pooled SMD was − 1.328 (95% CI: -2.550 to -0.107), indicating a significant reduction in fatigue levels (*p* = 0.033) (Fig. 7). The analysis demonstrated high heterogeneity (I² = 95.7%), suggesting substantial variability in the effect sizes across the included studies. Publication bias was not assessed due to limitation in the number of studies.

### Pain

**Fig. 8:**
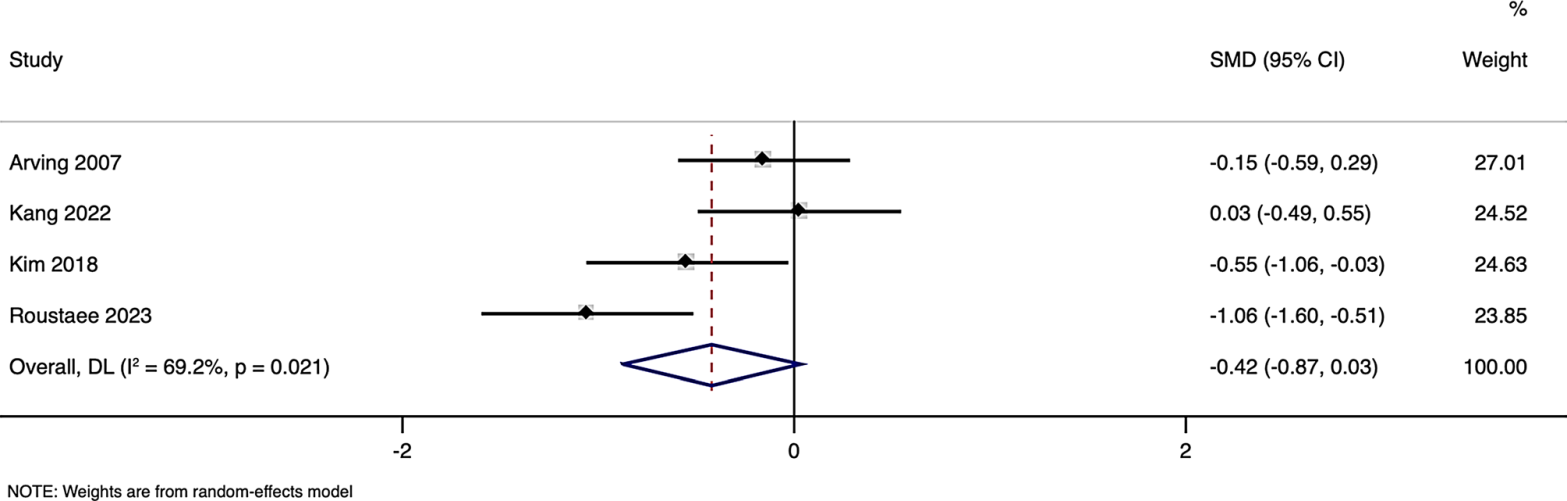
Forest plot showing the effectiveness of nurse-led interventions for pain amongst breast cancer patients. *Footnotes*: DL, DerSimonian–Laird random‑effects model; SMD, standardized mean difference; CI, confidence interval; %, inverse‑variance weight of each study. Squares represent study‑specific effect estimates (SMD) and their size is proportional to the study weight; Horizontal lines indicate the 95% confidence intervals; The solid vertical line denotes the line of no effect (SMD = 0); The dashed vertical line indicates the pooled effect estimate; Diamonds represent the overall pooled effect size with their width corresponding to the 95% confidence interval; Black markers and lines show individual studies; the blue diamond shows the pooled random‑effects estimate

This meta-analysis assessed the effect of nurse-led interventions on pain management in 257 participants across four studies. The pooled SMD was − 0.421 (95% CI: -0.874 to 0.031), indicating a trend towards pain reduction that approached but did not reach statistical significance (*p* = 0.068) (Fig. 8). The studies exhibited considerable heterogeneity (I² = 69.2%), suggesting variations in intervention effects. Publication bias was not assessed due to limitation in the number of studies.

### Dyspnea

**Fig. 9:**
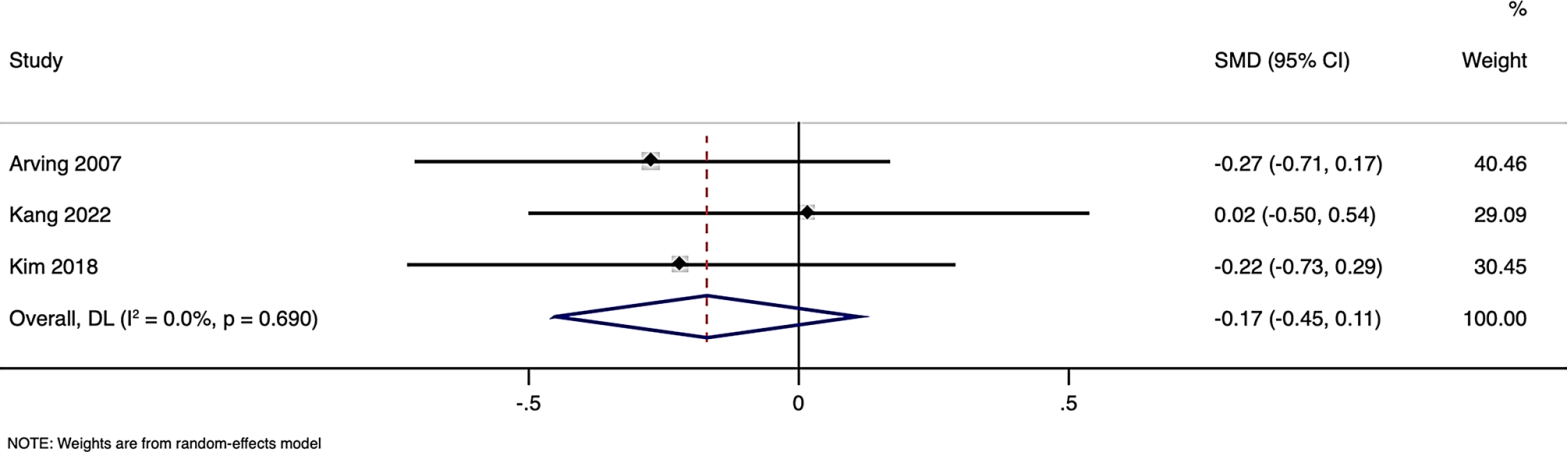
Forest plot showing the effectiveness of nurse-led interventions for dyspnea amongst breast cancer patients. *Footnotes*: DL, DerSimonian–Laird random‑effects model; SMD, standardized mean difference; CI, confidence interval; %, inverse‑variance weight of each study. Squares represent study‑specific effect estimates (SMD) and their size is proportional to the study weight; Horizontal lines indicate the 95% confidence intervals; The solid vertical line denotes the line of no effect (SMD = 0); The dashed vertical line indicates the pooled effect estimate; Diamonds represent the overall pooled effect size with their width corresponding to the 95% confidence interval; Black markers and lines show individual studies; the blue diamond shows the pooled random‑effects estimate

This meta-analysis evaluated the effect of nurse-led interventions on dyspnea across three studies involving 197 participants. The pooled SMD was − 0.171 (95% CI: -0.451 to 0.110), showing a non-significant reduction in dyspnea symptoms (*p* = 0.233) (Fig. 9). Heterogeneity among the studies was minimal (I² = 0.0%), indicating consistent effects of the interventions across different studies. Publication bias was not assessed due to limitation in the number of studies.

### Nausea/vomiting

**Fig. 10:**
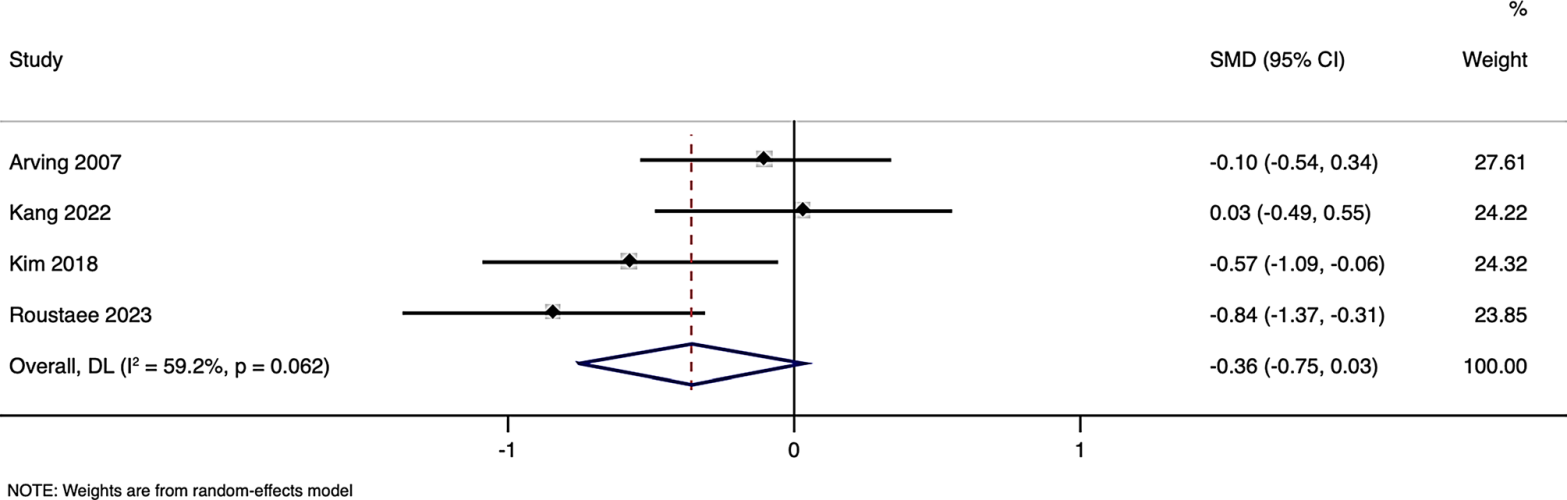
Forest plot showing the effectiveness of nurse-led interventions for nausea/vomiting amongst breast cancer patients. *Footnotes*: DL, DerSimonian–Laird random‑effects model; SMD, standardized mean difference; CI, confidence interval; %, inverse‑variance weight of each study. Squares represent study‑specific effect estimates (SMD) and their size is proportional to the study weight; Horizontal lines indicate the 95% confidence intervals; The solid vertical line denotes the line of no effect (SMD = 0); The dashed vertical line indicates the pooled effect estimate; Diamonds represent the overall pooled effect size with their width corresponding to the 95% confidence interval; Black markers and lines show individual studies; the blue diamond shows the pooled random‑effects estimate

This meta-analysis assessed the impact of interventions on nausea across four studies involving 257 participants. The pooled SMD was − 0.359, with 95% CI ranging from − 0.750 to 0.031, indicating a trend toward reduced nausea symptoms, although not statistically significant (*p* = 0.072) (Fig. 10). Heterogeneity among the studies was moderate, with an I² of 59.2%, suggesting some variability in the effect sizes across studies. Publication bias was not assessed due to limitation in the number of studies.

### Additional analysis

Leave-one out sensitivity analysis was performed for each of the above outcomes to see small or single study effects and robustness of the results (Supplementary Figs. 3 to 11). However, none of the outcomes showed any change with respect to direction or magnitude of the pooled effect size. However, subgroup analysis was not possible due to limited number of studies across different subgroups for each of the outcomes.

## Discussion

This meta-analysis examined the effects of nurse-led interventions on various aspects of quality of life in breast cancer patients, including global health status, functional status, symptom scores, role functioning, emotional functioning, fatigue, pain, dyspnea, and nausea/vomiting. The findings revealed modest improvements in global health status (SMD = 0.265) and functional status (SMD = 0.241), though these were not statistically significant. Significant reductions were observed in fatigue (SMD = -1.328, *p* = 0.033), while other outcomes such as symptom scores, role functioning, emotional functioning, pain, dyspnea, and nausea/vomiting showed non-significant trends towards improvement. Substantial heterogeneity was noted across most outcomes, indicating variability in the effects of the interventions.

The findings of this meta-analysis are consistent with previous research that has highlighted the potential benefits of nurse-led interventions in enhancing the quality of life for breast cancer patients [6, 7, 12]. Prior studies have demonstrated similar modest improvements in global health status and functional outcomes. For instance, a systematic review by [12] found that psychoeducational interventions led by nurses improved various quality of life domains for cancer patients. Similarly, a review by [6] suggested that nurse-led interventions could positively affect emotional and psychological well-being, although the magnitude of these effects varied [7]. These findings emphasise that statistically detectable gains in QoL may still be below MCID thresholds, underscoring the need to prioritise intervention components and delivery strategies that yield changes that are both statistically and clinically meaningful.

However, our findings also diverge from some previous studies that reported more pronounced benefits. For example, a meta-analysis by [13] found significant improvements in symptom management and emotional functioning following nurse-led interventions. The variability in our findings may be attributed to differences in study design, sample sizes, types of interventions, and patient populations across the included studies [13].

Several mechanisms may explain the observed effects of nurse-led interventions on the quality of life in breast cancer patients. Firstly, nurse-led interventions often provide personalized and continuous support, which can enhance patients’ sense of control and reduce anxiety and depression. This personalized care is particularly important in managing complex symptoms and side effects associated with breast cancer treatment, such as fatigue and pain [6, 7, 12].

Additionally, nurse-led interventions frequently include educational components that empower patients with knowledge about their condition and treatment, enabling them to manage symptoms more effectively. Education and counselling can also help patients develop coping strategies, which may improve their emotional and functional well-being [7, 12].

Differences between our findings and earlier reviews likely reflect variation across four methodological and clinical dimensions. First, measurement and analysis choices: we meta-analysed heterogeneous QoL instruments using standardized mean differences (Hedges g), reported τ² and 95% prediction intervals, and interpreted effects against instrument-specific MCIDs or a distribution-based proxy. Several prior syntheses pooled instrument-specific means without standardization, emphasised statistical significance over clinical thresholds, combined global QoL with symptom scales, or did not report τ²/prediction intervals—approaches that can yield larger summary effects and narrower uncertainty. Second, intervention definitions and fidelity: we applied a stricter, operational definition of “nurse-led” (nurse as lead provider and ≥ 50% nurse-delivered contact time), excluding multidisciplinary or psychologist-led programmes without nurse leadership; we also coded intensity, modality (in-person/telephone/telehealth), and core components. Earlier reviews often grouped diverse “supportive care” models together, which can inflate apparent benefits. Third, populations, comparators, and timing: our included trials span different treatment phases (peri-chemotherapy, radiotherapy, survivorship) and healthcare contexts; baseline QoL varied widely, and usual-care comparators ranged from minimal to enhanced follow-up, with occasional contamination. Reviews focused on narrower phases, or with less intensive comparators, tend to observe larger effects. Fourth, study quality and small-study effects: our dataset includes more recent trials with clearer allocation methods and intention-to-treat analyses but also broader geographical diversity; where asymmetry suggested small-study effects, we interpreted results cautiously. Collectively, these factors plausibly explain why our pooled effects are statistically detectable yet often below MCID thresholds, whereas some earlier reviews reported larger improvements.

Our findings extend the nursing literature in three practice-relevant ways. First, by interpreting pooled standardized effects against minimal clinically important difference (MCID) thresholds and reporting τ² and 95% prediction intervals, we show that apparent statistical improvements often remain clinically marginal for global and functional quality of life, while the fatigue reduction is both statistically significant and potentially practice-meaningful. This distinction matters for bedside decision-making and quality dashboards, where resource allocation should prioritise symptom domains most likely to yield patient-perceived benefit. Second, we operationalised “nurse-led” using a priori criteria (nurse as lead provider; ≥50% nurse-delivered contact time; protocolised components). This sharper definition clarifies which models of nursing actually drive effect, separating them from generic “supportive care” bundles that dilute signal and complicate implementation. Third, by back-translating SMDs where feasible and triangulating with MCIDs, we provide a pragmatic bridge from meta-analytic metrics to outcomes nurses can track (e.g., EORTC QLQ-C30 fatigue and global health scores) within routine clinics.

Mechanistically, three pathways likely underpin the observed pattern of benefits. Capability building (self-management coaching, adherence support) and emotional regulation (brief psychoeducation, problem-solving, relaxation) plausibly reduce treatment-related fatigue more than they alter broad global QOL composites, which are multidetermined and slower to shift. Continuity and coordination delivered by nurse navigators (proactive follow-up, anticipatory symptom titration) may compress symptom duration and intensity, generating cumulative improvements that are not fully captured by single end-point global scales. These mechanisms align with contemporary nursing models (e.g., symptom science, navigation/coordination) and suggest that dose (contact frequency), timing (peri-chemotherapy peaks), and fidelity (adherence to algorithms) are leverage points for practice optimisation.

Heterogeneity in effects should not be read as “ineffectiveness,” but as actionable variability tied to intervention design and context. Our extraction shows wide dispersion in components (psychoeducational vs. exercise-rehabilitation vs. medication self-management), delivery mode (in-person vs. telehealth), intensity (two sessions to multi-week programmes), and patient timing (active treatment vs. survivorship). This implies that services should match components to need: fatigue-dominant phenotypes may benefit most from nurse-supervised rehabilitation plus self-management coaching; high-distress phenotypes may require brief stepped psycho-oncology integrated within nurse-led follow-up. Future trials should prespecify these phenotypes and test component efficacy rather than monolithic bundles.

For clinical services, we translate the meta-analytic signal into a three-step implementation schema: (1) Screen and stratify at each visit using brief tools (e.g., EORTC QLQ-C30 fatigue/emotional items) embedded in nursing workflow; (2) Deliver a protocolised, nurse-led core (education + self-management + early titration/coordination) with optional modules (exercise coaching; brief relaxation; medication self-management) triggered by thresholds; and (3) Track response against instrument-specific MCIDs to guide step-up to multidisciplinary care. This schema is feasible in outpatient chemotherapy and survivorship clinics and compatible with tele-nursing for rural/underserved populations [20].

### Limitations of this review

Several limitations must also be acknowledged. There was significant heterogeneity in study design, intervention types, and the specific quality-of-life outcomes assessed, which may limit the generalizability of our findings. In addition, some outcomes were evaluated based on a limited number of studies, potentially reducing the robustness of the conclusions for those specific endpoints. Despite our efforts to detect and account for publication bias, its presence cannot be entirely ruled out, particularly for outcomes where funnel plot asymmetry was observed. Lastly, the variability in risk-of-bias ratings among the included studies further underscores the need for cautious interpretation of the pooled estimates. It is important to note that the protocol for this study was not registered in a public database prior to commencement. We acknowledge this as a limitation, as the lack of prospective registration prevents comparison between planned and reported outcomes, thereby introducing a potential risk of selective reporting bias. However, to minimize this risk, we adhered strictly to the PRISMA guidelines and have reported all outcomes analyzed as outlined in our methodology section.

## Conclusion

Nurse-led interventions probably confer small, directionally favorable improvements in health-related quality of life for people with breast cancer; however, effects are often near or below minimal clinically important difference thresholds, with substantial variation across formats and contexts. Future trials should standardize intervention components and outcome measures (including symptom-specific instruments and MCID reporting), ensure adequate sample size and fidelity, and compare against clearly defined usual care.

## Supplementary Information

Below is the link to the electronic supplementary material.

Supplementary Material 1

Supplementary Material 2: Supplementary Fig.1: Funnel plot for global health status/overall quality of life. *Footnote*: Filled circles represent individual study effect sizes (SMD). The vertical solid line indicates the pooled effect size, and the dashed lines represent pseudo 95% confidence limits around the pooled effect.

Supplementary Material 3: Supplementary Fig.2: Funnel plot for functional status. *Footnote*: Filled circles represent individual study effect sizes (SMD). The vertical solid line indicates the pooled effect size, and the dashed lines represent pseudo 95% confidence limits around the pooled effect.

Supplementary Material 4: Supplementary Fig.3: Sensitivity analysis plot for global health status/overall quality of life. *Footnote*: Open circles represent the pooled standardized mean difference (SMD) after omitting the named study. Horizontal dotted lines show the corresponding 95% confidence intervals. The central vertical solid line indicates the overall pooled SMD including all studies, and outer vertical lines indicate the 95% confidence interval of this overall estimate.

Supplementary Material 5: Supplementary Fig.4: Sensitivity analysis plot for functional status. *Footnote*: Open circles represent the pooled standardized mean difference (SMD) after omitting the named study. Horizontal dotted lines show the corresponding 95% confidence intervals. The central vertical solid line indicates the overall pooled SMD including all studies, and outer vertical lines indicate the 95% confidence interval of this overall estimate.

Supplementary Material 6: Supplementary Fig.5: Sensitivity analysis plot for symptom score. *Footnote*: Open circles represent the pooled standardized mean difference (SMD) after omitting the named study. Horizontal dotted lines show the corresponding 95% confidence intervals. The central vertical solid line indicates the overall pooled SMD including all studies, and outer vertical lines indicate the 95% confidence interval of this overall estimate.

Supplementary Material 7: Supplementary Fig.6: Sensitivity analysis plot for role functioning. *Footnote*: Open circles represent the pooled standardized mean difference (SMD) after omitting the named study. Horizontal dotted lines show the corresponding 95% confidence intervals. The central vertical solid line indicates the overall pooled SMD including all studies, and outer vertical lines indicate the 95% confidence interval of this overall estimate.

Supplementary Material 8: Supplementary Fig.7: Sensitivity analysis plot for emotional functioning. *Footnote*: Open circles represent the pooled standardized mean difference (SMD) after omitting the named study. Horizontal dotted lines show the corresponding 95% confidence intervals. The central vertical solid line indicates the overall pooled SMD including all studies, and outer vertical lines indicate the 95% confidence interval of this overall estimate.

Supplementary Material 9: Supplementary Fig.8: Sensitivity analysis plot for fatigue. *Footnote*: Open circles represent the pooled standardized mean difference (SMD) after omitting the named study. Horizontal dotted lines show the corresponding 95% confidence intervals. The central vertical solid line indicates the overall pooled SMD including all studies, and outer vertical lines indicate the 95% confidence interval of this overall estimate.

Supplementary Material 10: Supplementary Fig.9: Sensitivity analysis plot for pain. *Footnote*: Open circles represent the pooled standardized mean difference (SMD) after omitting the named study. Horizontal dotted lines show the corresponding 95% confidence intervals. The central vertical solid line indicates the overall pooled SMD including all studies, and outer vertical lines indicate the 95% confidence interval of this overall estimate.

Supplementary Material 11: Supplementary Fig.10: Sensitivity analysis plot for dyspnea. *Footnote*: Open circles represent the pooled standardized mean difference (SMD) after omitting the named study. Horizontal dotted lines show the corresponding 95% confidence intervals. The central vertical solid line indicates the overall pooled SMD including all studies, and outer vertical lines indicate the 95% confidence interval of this overall estimate.

Supplementary Material 12: Supplementary Fig.11: Sensitivity analysis plot for nausea/vomiting. *Footnote*: Open circles represent the pooled standardized mean difference (SMD) after omitting the named study. Horizontal dotted lines show the corresponding 95% confidence intervals. The central vertical solid line indicates the overall pooled SMD including all studies, and outer vertical lines indicate the 95% confidence interval of this overall estimate.

## Data Availability

Data will be made available upon reasonable request from authors.

## Abbreviations

CI: Confidence interval
FACT: Functional Assessment of Cancer Therapy
MCID: Minimal clinically important differences
POMS: Profile of Mood States
PRISMA: Preferred Reporting Items for Systematic Review and Meta-Analysis
QoL: Quality of life
SD: Standrad deviation
SMD: Standardized mean difference
