# Supplementary material for: Nurse-led intervention for improving quality of life of breast cancer patients: systematic review and meta-analysis
Source: BMC Nurs. 2026 May 14;25:446. doi: 10.1186/s12912-026-04505-2 (PMC13379969; doi:10.1186/s12912-026-04505-2)
Supplement: Supplementary file 1 — Supplementary Material 1 [file 12912_2026_4505_MOESM1_ESM.pdf]

Supplementary Appendix:

Search strategy:

PubMed:

(("Breast Neoplasms"[Mesh] OR "breast cancer"[tiab] OR "breast cancers"[tiab] OR breast neoplasm*[tiab] OR breast tumor*[tiab] OR breast tumour*[tiab] OR mammary carcinoma*[tiab] OR "carcinoma of the breast"[tiab] OR "breast malignan*"[tiab] OR "breast adenocarcinoma*"[tiab]) AND ("Nursing"[Mesh] OR "Nurses"[Mesh] OR "Nursing Care"[Mesh] OR "Nurse's Role"[Mesh] OR "Case Management"[Mesh] OR "Patient Navigation"[Mesh] OR nurse-led[tiab] OR "nurse led"[tiab] OR "nurse-directed"[tiab] OR "nurse managed"[tiab] OR "nurse-managed"[tiab] OR "nurse-delivered"[tiab] OR "nurse delivered"[tiab] OR "nurse-administered"[tiab] OR "nurse administered"[tiab] OR "nursing intervention*"[tiab] OR "nursing care"[tiab] OR "oncology nurse*"[tiab] OR "cancer nurse*"[tiab] OR "breast care nurse*"[tiab] OR "specialist breast care nurse*"[tiab] OR "nurse navigator*"[tiab] OR "nurse-led clinic*"[tiab] OR "follow-up nurse*"[tiab] OR "telephone nurs*"[tiab] OR telenurs*[tiab] OR "tele-nurs*"[tiab] OR "telehealth nurs*"[tiab] OR "case manag*"[tiab] OR "care coordinat*"[tiab] OR psychoeduc*[tiab] OR counsel*[tiab] OR "self-manag*"[tiab] OR "survivorship care"[tiab] OR "patient support"[tiab]) AND ("Quality of Life"[Mesh] OR "quality of life"[tiab] OR "health-related quality of life"[tiab] OR HRQoL[tiab] OR QoL[tiab] OR "global health"[tiab] OR "overall quality of life"[tiab] OR "functional status"[tiab] OR "role function*"[tiab] OR "emotional function*"[tiab] OR fatigue[tiab] OR pain[tiab] OR dyspn*[tiab] OR nausea[tiab] OR vomiting[tiab] OR symptom*[tiab] OR wellbeing[tiab] OR "well-being"[tiab] OR EORTC[tiab] OR "EORTC QLQ-C30"[tiab] OR "QLQ-C30"[tiab] OR "QLQ C30"[tiab] OR "FACT-B"[tiab] OR "Functional Assessment of Cancer Therapy-Breast"[tiab] OR "Hospital

Anxiety and Depression Scale"[tiab] OR HADS[tiab] OR "EQ-5D"[tiab] OR "SF-36"[tiab] OR SF36[tiab] OR WHOQOL[tiab] OR PROMIS[tiab])) AND (randomized controlled trial[pt] OR randomized[tiab] OR randomised[tiab] OR RCT[tiab] OR "clinical trial"[pt] OR trial[tiab]) AND ("1956/01/01"[Date - Publication] : "2024/10/31"[Date - Publication])

Scopus:

(TITLE-ABS-KEY((breast W/1 (cancer OR neoplasm* OR tumor* OR tumour* OR carcinoma* OR malignan* OR adenocarcinoma*)) AND (nurs* W/3 (led OR managed OR deliver* OR administered OR direct* OR intervention* OR navigator* OR "care coordination" OR "case management" OR psychoeduc* OR counsel* OR "self-management" OR education OR "follow-up" OR "follow up" OR clinic* OR telephone OR telehealth OR telenurs* OR "tele-nurs*")) AND ("quality of life" OR HRQoL OR QoL OR "global health" OR "functional status" OR "role function*" OR "emotional function*" OR fatigue OR pain OR dyspn* OR nausea OR vomiting OR symptom* OR wellbeing OR "well-being" OR EORTC OR "EORTC QLQ-C30" OR "QLQ-C30" OR "QLQ C30" OR "FACT-B" OR "Hospital Anxiety and Depression Scale" OR HADS OR "EQ-5D" OR "SF-36" OR SF36 OR WHOQOL OR PROMIS) AND ((random* W/3 trial*) OR RCT))) AND (PUBYEAR > 1955 AND PUBYEAR < 2025)

Cochrane library:

([mh "Breast Neoplasms"] OR (breast NEXT (cancer OR neoplasm* OR tumor* OR tumour* OR carcinoma* OR malignan* OR adenocarcinoma*) OR "mammary carcinoma" OR "carcinoma of the breast" OR "breast malignan*" OR "breast adenocarcinoma*")) AND (([mh Nursing] OR [mh Nurses] OR [mh "Nursing Care"] OR [mh "Case Management"] OR [mh "Patient Navigation"]) OR (nurs* NEAR/3 (led OR managed OR deliver* OR administered OR direct* OR intervention* OR navigator* OR "care coordination" OR "case

management" OR psychoeduc* OR counsel* OR "self-management" OR education OR "follow-up" OR "follow up" OR clinic* OR telephone OR telehealth OR telenurs* OR "tele-nurs*"))) AND (([mh "Quality of Life"] OR "quality of life" OR HRQoL OR QoL OR "global health" OR "functional status" OR "role function*" OR "emotional function*" OR fatigue OR pain OR dyspn* OR nausea OR vomiting OR symptom* OR wellbeing OR "well-being" OR EORTC OR "EORTC QLQ-C30" OR "QLQ-C30" OR "QLQ C30" OR "FACT-B" OR "Hospital Anxiety and Depression Scale" OR HADS OR "EQ-5D" OR "SF-36" OR SF36 OR WHOQOL OR PROMIS)) in Trials

CINAHL:

(((MH "Breast Neoplasms+") OR (TI (breast N1 (cancer OR neoplasm* OR tumor* OR tumour* OR carcinoma* OR malignan* OR adenocarcinoma*)) OR AB (breast N1 (cancer OR neoplasm* OR tumor* OR tumour* OR carcinoma* OR malignan* OR adenocarcinoma*)))) AND ((MH "Nursing+" OR MH "Nursing Care+" OR MH "Nurses+" OR MH "Case Management+" OR MH "Patient Navigation+") OR (TI (nurs* N3 (led OR managed OR deliver* OR administered OR direct* OR intervention* OR navigator* OR "care coordination" OR "case management" OR psychoeduc* OR counsel* OR "self-management" OR education OR "follow-up" OR "follow up" OR clinic* OR telephone OR telehealth OR telenurs* OR "tele-nurs*")) OR AB (nurs* N3 (led OR managed OR deliver* OR administered OR direct* OR intervention* OR navigator* OR "care coordination" OR "case management" OR psychoeduc* OR counsel* OR "self-management" OR education OR "follow-up" OR "follow up" OR clinic* OR telephone OR telehealth OR telenurs* OR "tele-nurs*")))) AND ((MH "Quality of Life+") OR TI ("quality of life" OR HRQoL OR QoL OR "global health" OR "functional status" OR "role function*" OR "emotional function*" OR fatigue OR pain OR dyspn* OR nausea OR vomiting OR symptom* OR wellbeing OR "well-being" OR EORTC OR "EORTC QLQ-C30" OR "QLQ-C30" OR "QLQ C30" OR "FACT-B"

OR "Hospital Anxiety and Depression Scale" OR HADS OR "EQ-5D" OR "SF-36" OR SF36 OR WHOQOL OR PROMIS) OR AB ("quality of life" OR HRQoL OR QoL OR "global health" OR "functional status" OR "role function*" OR "emotional function*" OR fatigue OR pain OR dyspn* OR nausea OR vomiting OR symptom* OR wellbeing OR "well-being" OR EORTC OR "EORTC QLQ-C30" OR "QLQ-C30" OR "QLQ C30" OR "FACT-B" OR "Hospital Anxiety and Depression Scale" OR HADS OR "EQ-5D" OR "SF-36" OR SF36 OR WHOQOL OR PROMIS)) AND ((PT "Randomized Controlled Trial") OR TI (random* OR trial*) OR AB (random* OR trial*)))
