# Supplementary figures and images for: Nurse-led intervention for improving quality of life of breast cancer patients: systematic review and meta-analysis
Source: BMC Nurs. 2026 May 14;25:446. doi: 10.1186/s12912-026-04505-2 (PMC13379969; doi:10.1186/s12912-026-04505-2)

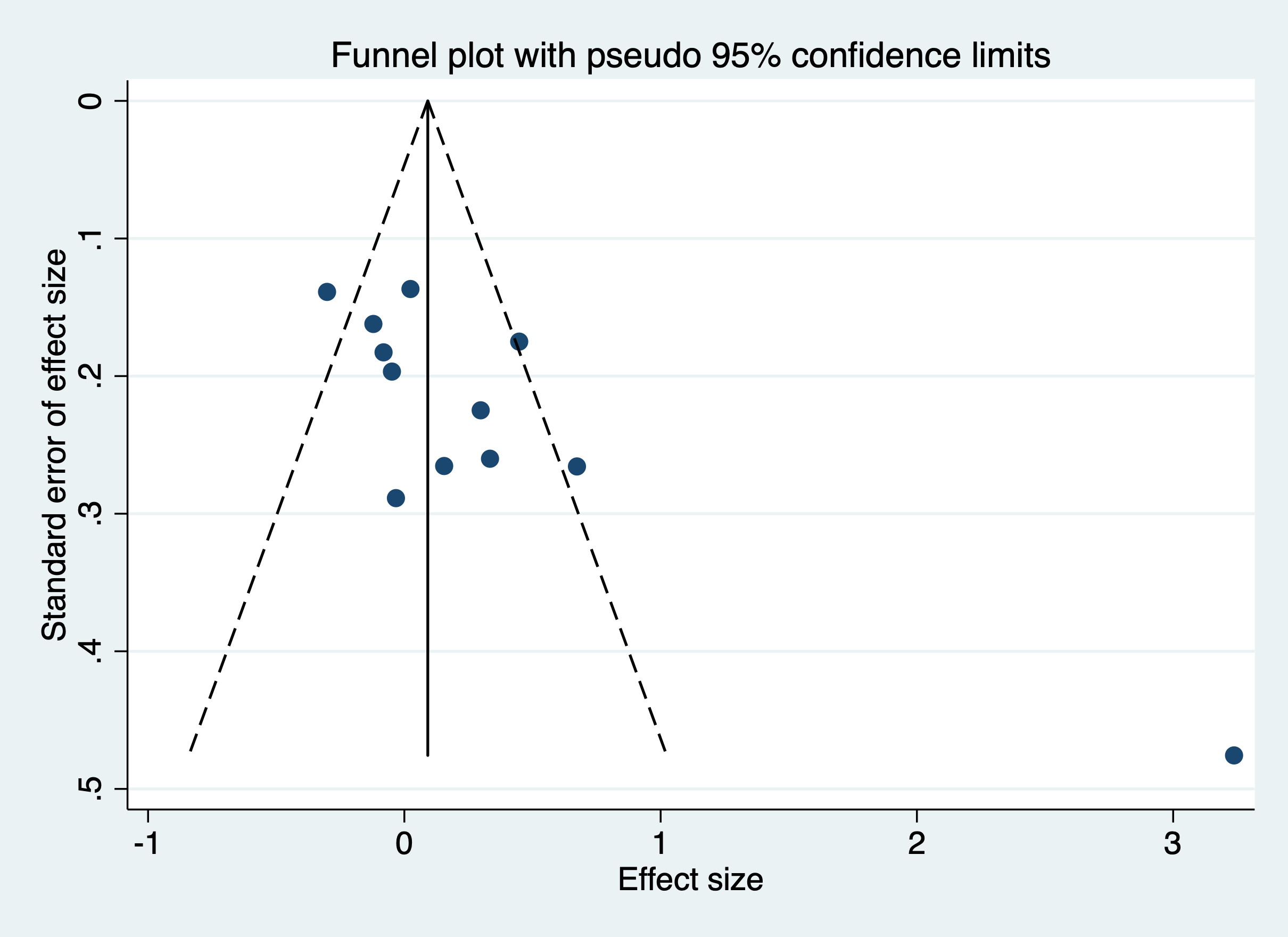

Supplement: Supplementary file 2 — Supplementary Material 2: Supplementary Fig.1: Funnel plot for global health status/overall quality of life. Footnote: Filled circles represent individual study effect sizes (SMD). The vertical solid line indicates the pooled effect size, and the dashed lines represent pseudo 95% confidence limits around the pooled effect. [file 12912_2026_4505_MOESM2_ESM.jpg]

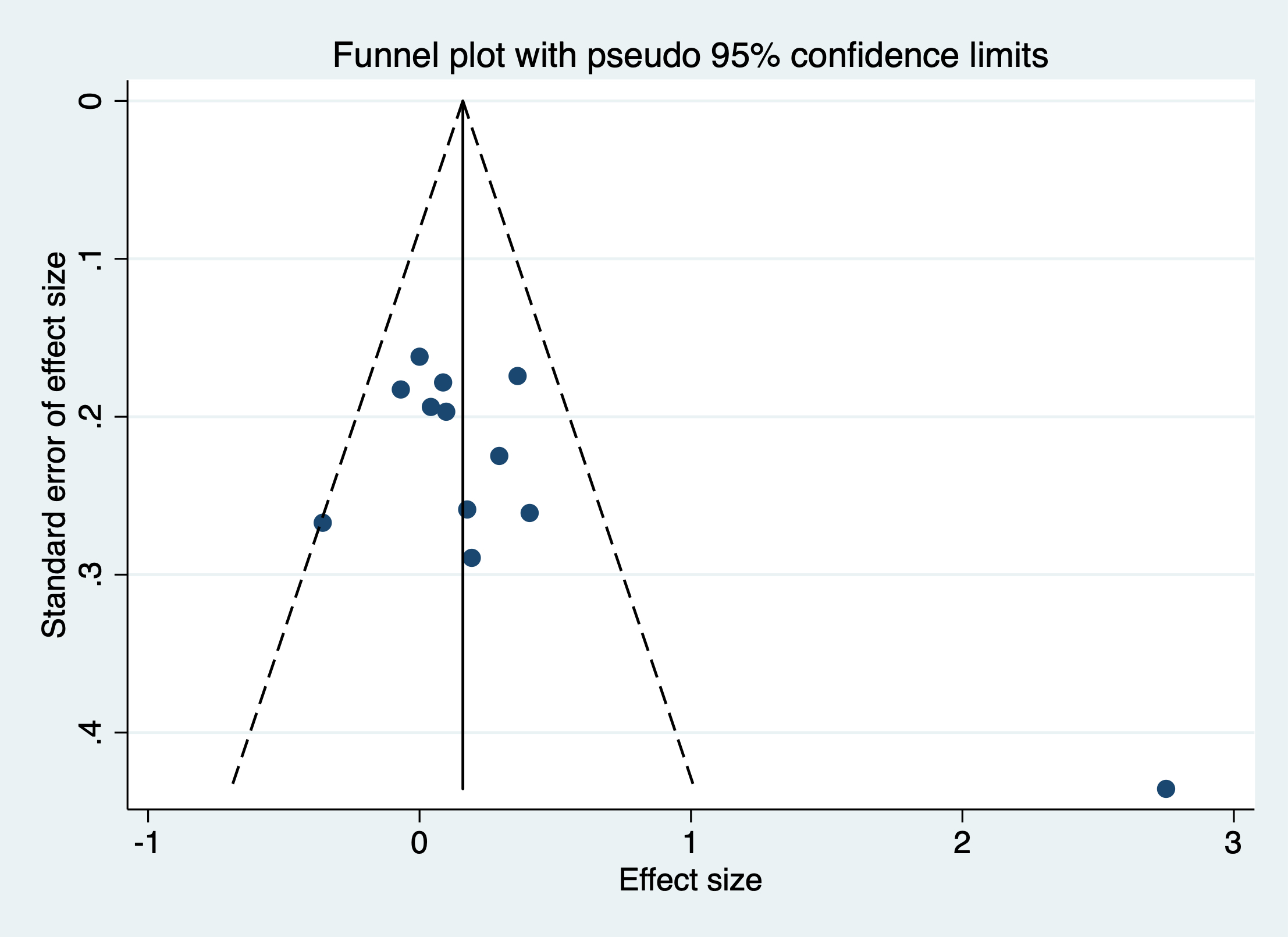

Supplement: Supplementary file 3 — Supplementary Material 3: Supplementary Fig.2: Funnel plot for functional status. Footnote: Filled circles represent individual study effect sizes (SMD). The vertical solid line indicates the pooled effect size, and the dashed lines represent pseudo 95% confidence limits around the pooled effect. [file 12912_2026_4505_MOESM3_ESM.jpg]

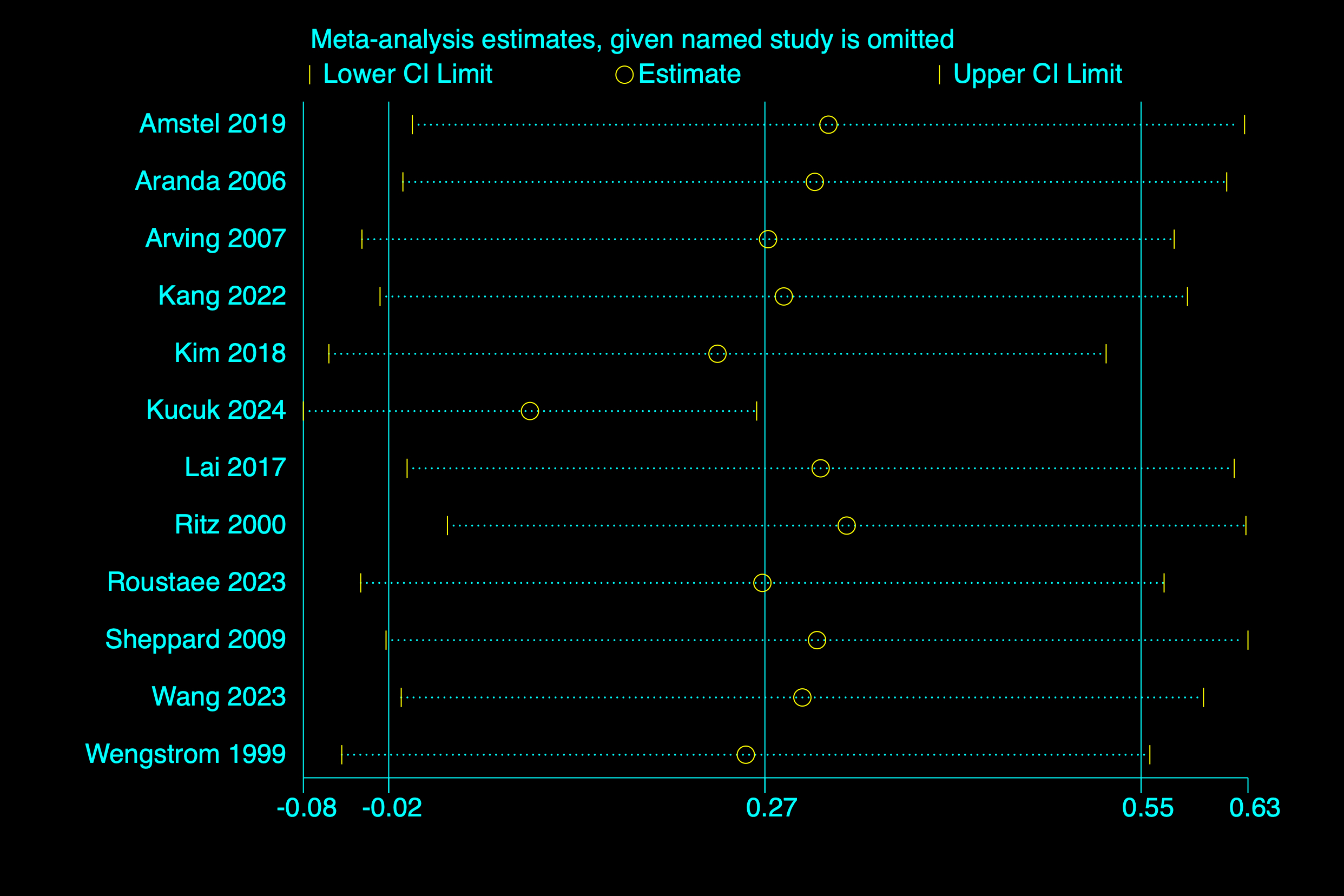

Supplement: Supplementary file 4 — Supplementary Material 4: Supplementary Fig.3: Sensitivity analysis plot for global health status/overall quality of life. Footnote: Open circles represent the pooled standardized mean difference (SMD) after omitting the named study. Horizontal dotted lines show the corresponding 95% confidence intervals. The central vertical solid line indicates the overall pooled SMD including all studies, and outer vertical lines indicate the 95% confidence interval of this overall estimate. [file 12912_2026_4505_MOESM4_ESM.jpg]

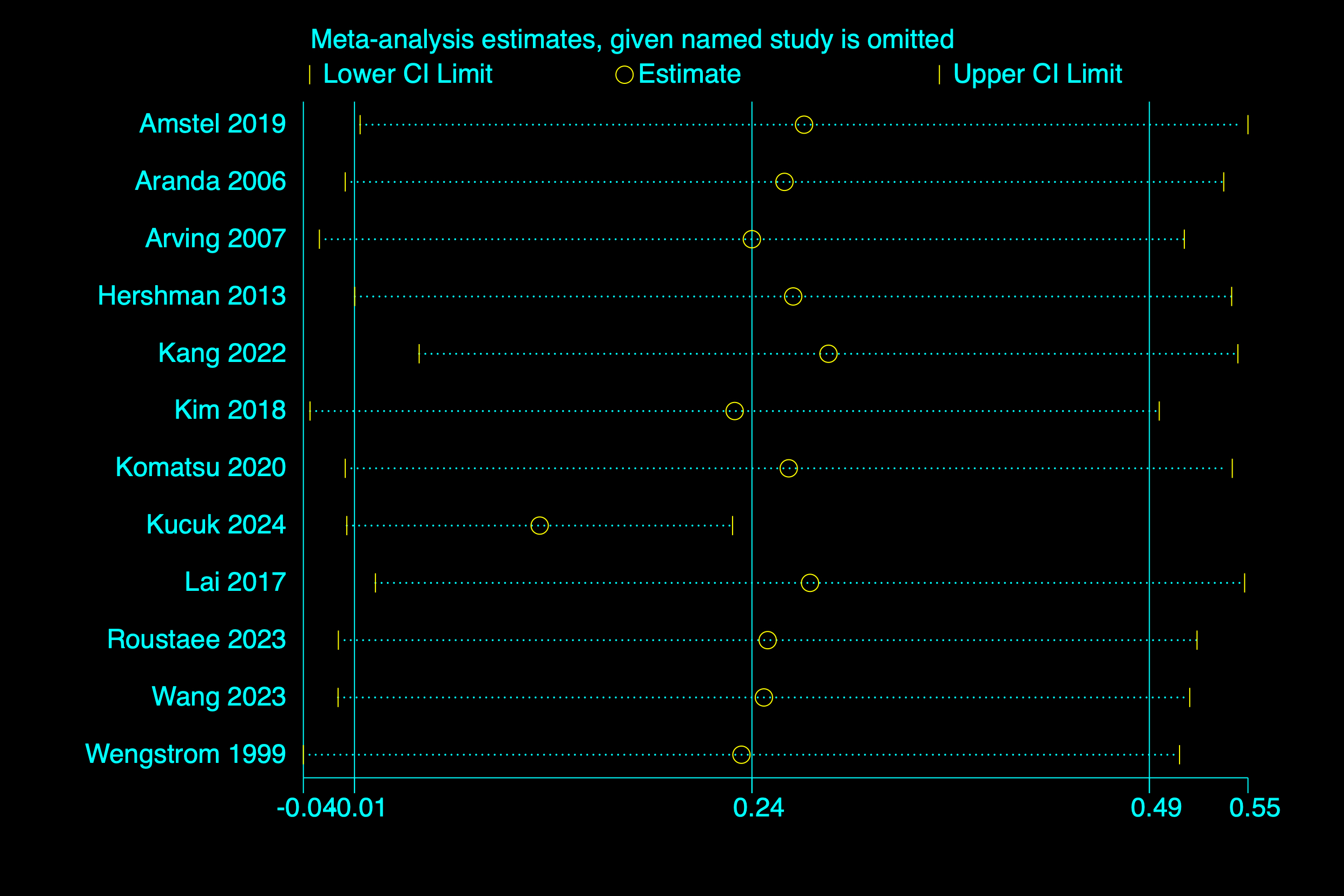

Supplement: Supplementary file 5 — Supplementary Material 5: Supplementary Fig.4: Sensitivity analysis plot for functional status. Footnote: Open circles represent the pooled standardized mean difference (SMD) after omitting the named study. Horizontal dotted lines show the corresponding 95% confidence intervals. The central vertical solid line indicates the overall pooled SMD including all studies, and outer vertical lines indicate the 95% confidence interval of this overall estimate. [file 12912_2026_4505_MOESM5_ESM.jpg]

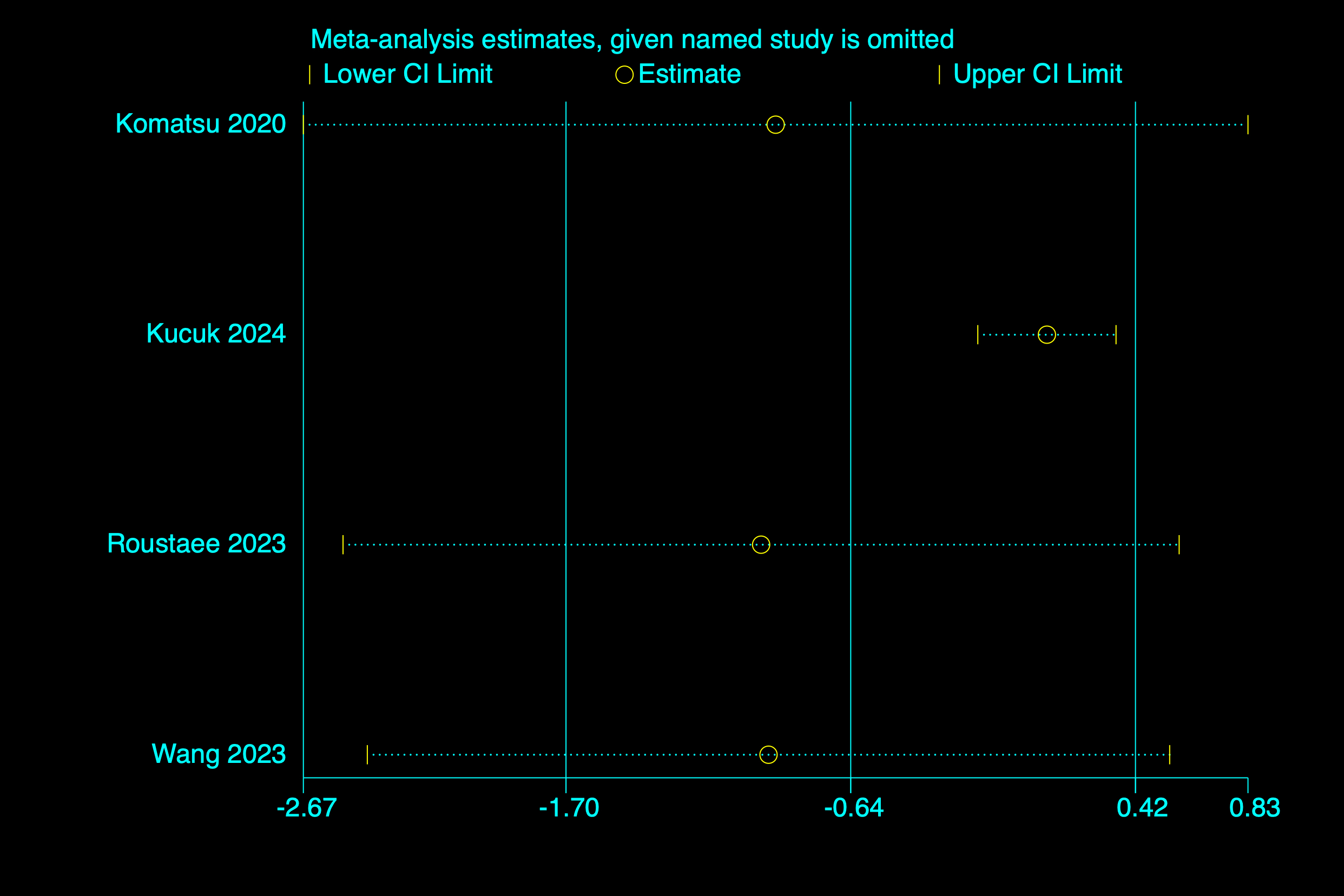

Supplement: Supplementary file 6 — Supplementary Material 6: Supplementary Fig.5: Sensitivity analysis plot for symptom score. Footnote: Open circles represent the pooled standardized mean difference (SMD) after omitting the named study. Horizontal dotted lines show the corresponding 95% confidence intervals. The central vertical solid line indicates the overall pooled SMD including all studies, and outer vertical lines indicate the 95% confidence interval of this overall estimate. [file 12912_2026_4505_MOESM6_ESM.jpg]

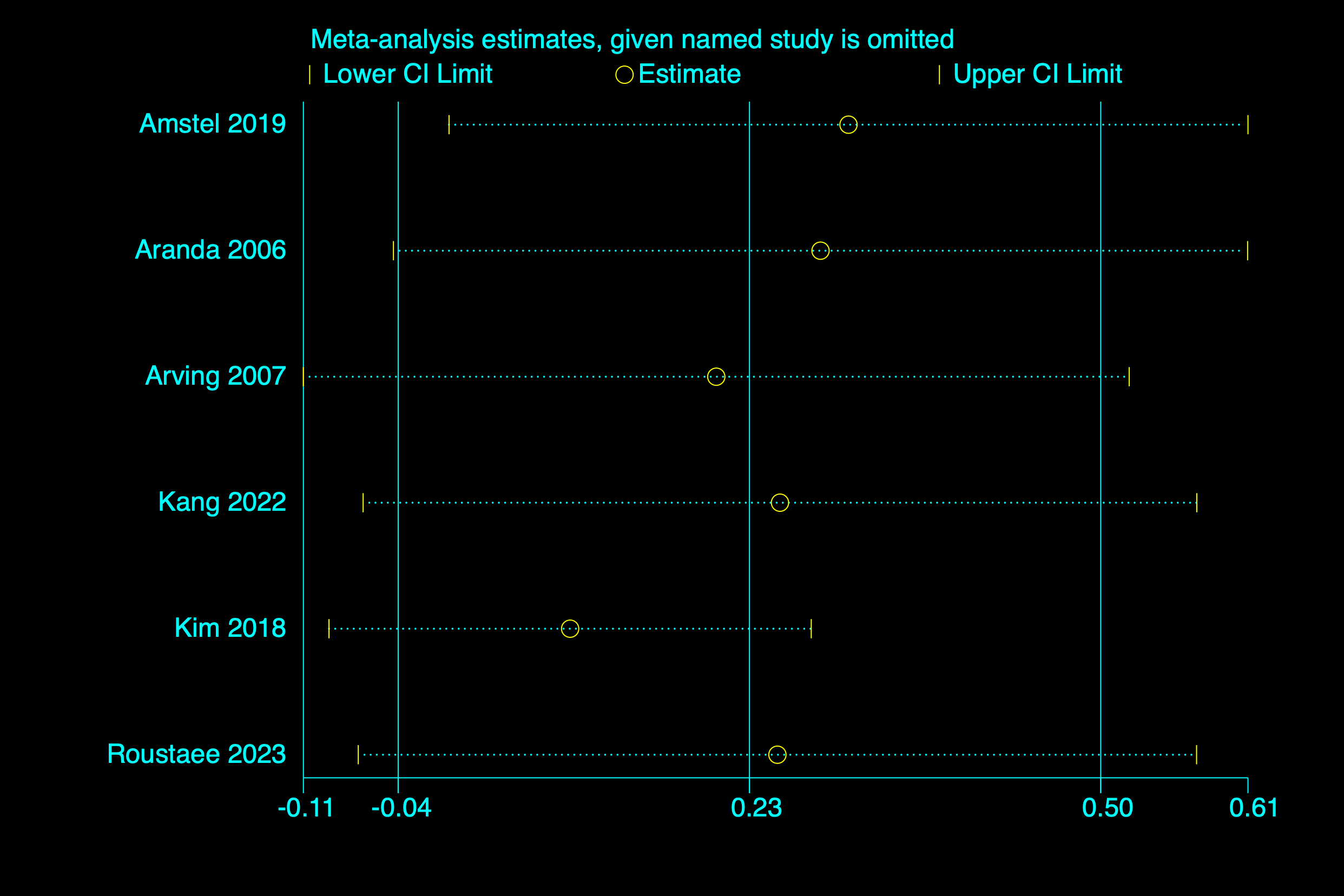

Supplement: Supplementary file 7 — Supplementary Material 7: Supplementary Fig.6: Sensitivity analysis plot for role functioning. Footnote: Open circles represent the pooled standardized mean difference (SMD) after omitting the named study. Horizontal dotted lines show the corresponding 95% confidence intervals. The central vertical solid line indicates the overall pooled SMD including all studies, and outer vertical lines indicate the 95% confidence interval of this overall estimate. [file 12912_2026_4505_MOESM7_ESM.jpg]

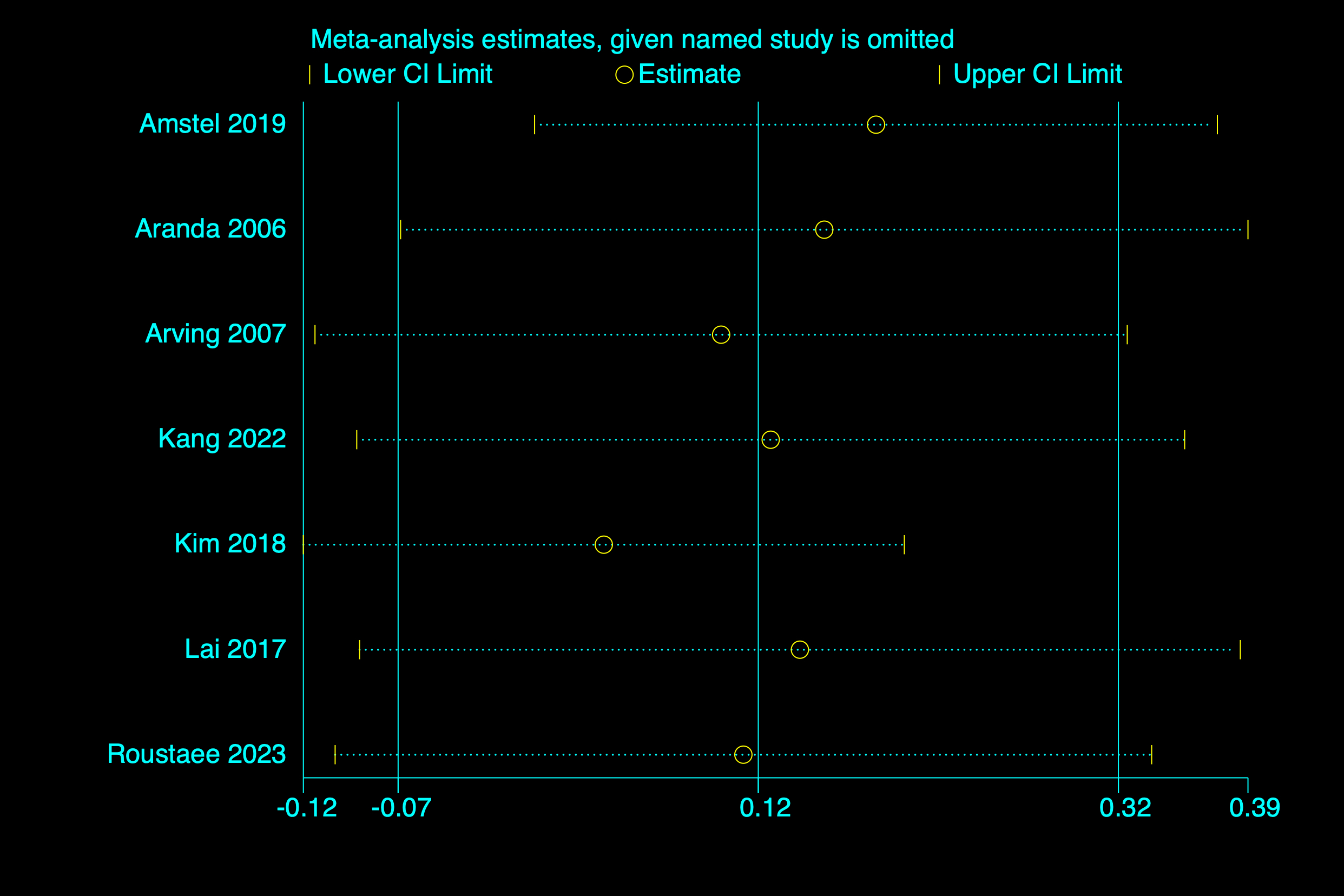

Supplement: Supplementary file 8 — Supplementary Material 8: Supplementary Fig.7: Sensitivity analysis plot for emotional functioning. Footnote: Open circles represent the pooled standardized mean difference (SMD) after omitting the named study. Horizontal dotted lines show the corresponding 95% confidence intervals. The central vertical solid line indicates the overall pooled SMD including all studies, and outer vertical lines indicate the 95% confidence interval of this overall estimate. [file 12912_2026_4505_MOESM8_ESM.jpg]

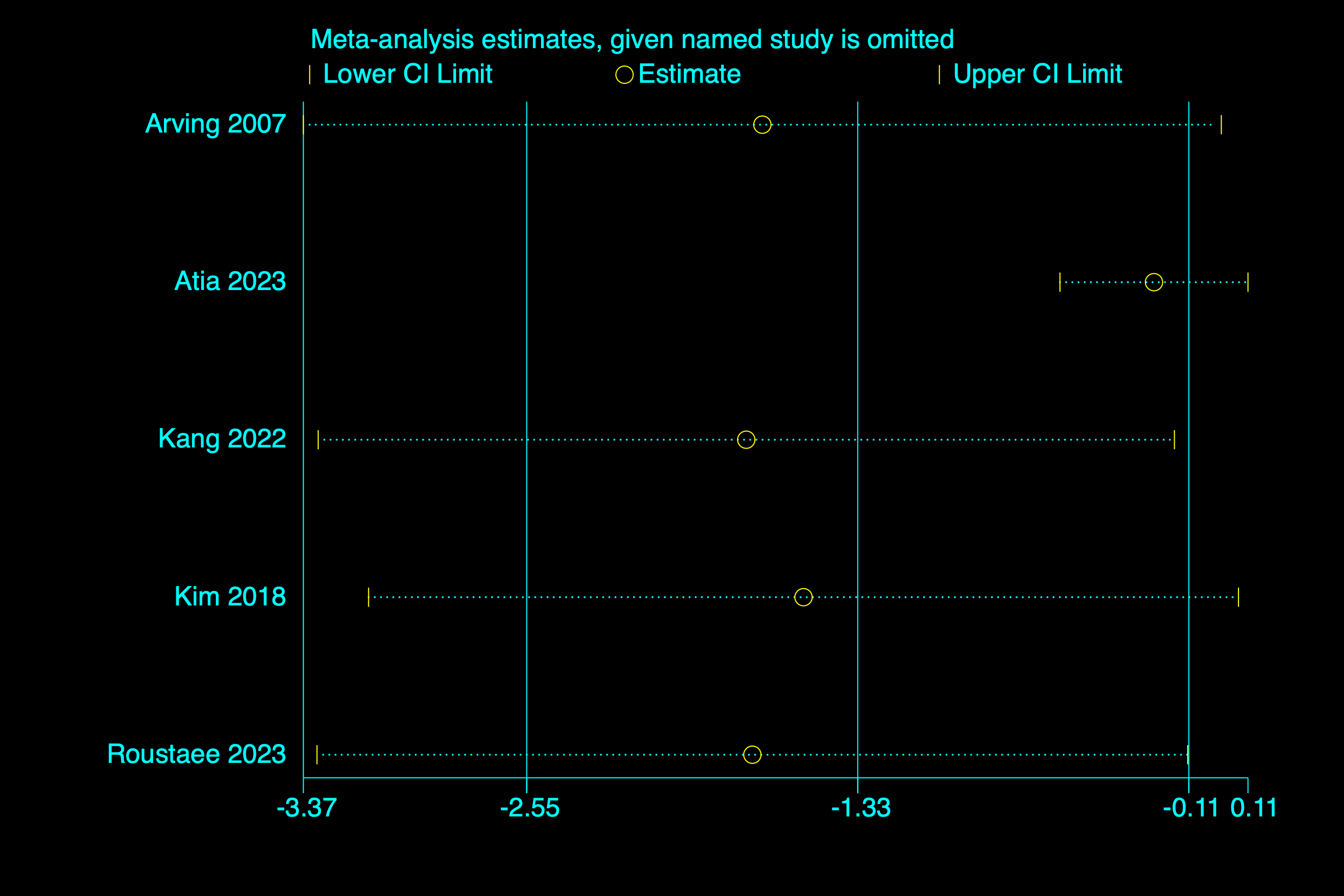

Supplement: Supplementary file 9 — Supplementary Material 9: Supplementary Fig.8: Sensitivity analysis plot for fatigue. Footnote: Open circles represent the pooled standardized mean difference (SMD) after omitting the named study. Horizontal dotted lines show the corresponding 95% confidence intervals. The central vertical solid line indicates the overall pooled SMD including all studies, and outer vertical lines indicate the 95% confidence interval of this overall estimate. [file 12912_2026_4505_MOESM9_ESM.jpg]

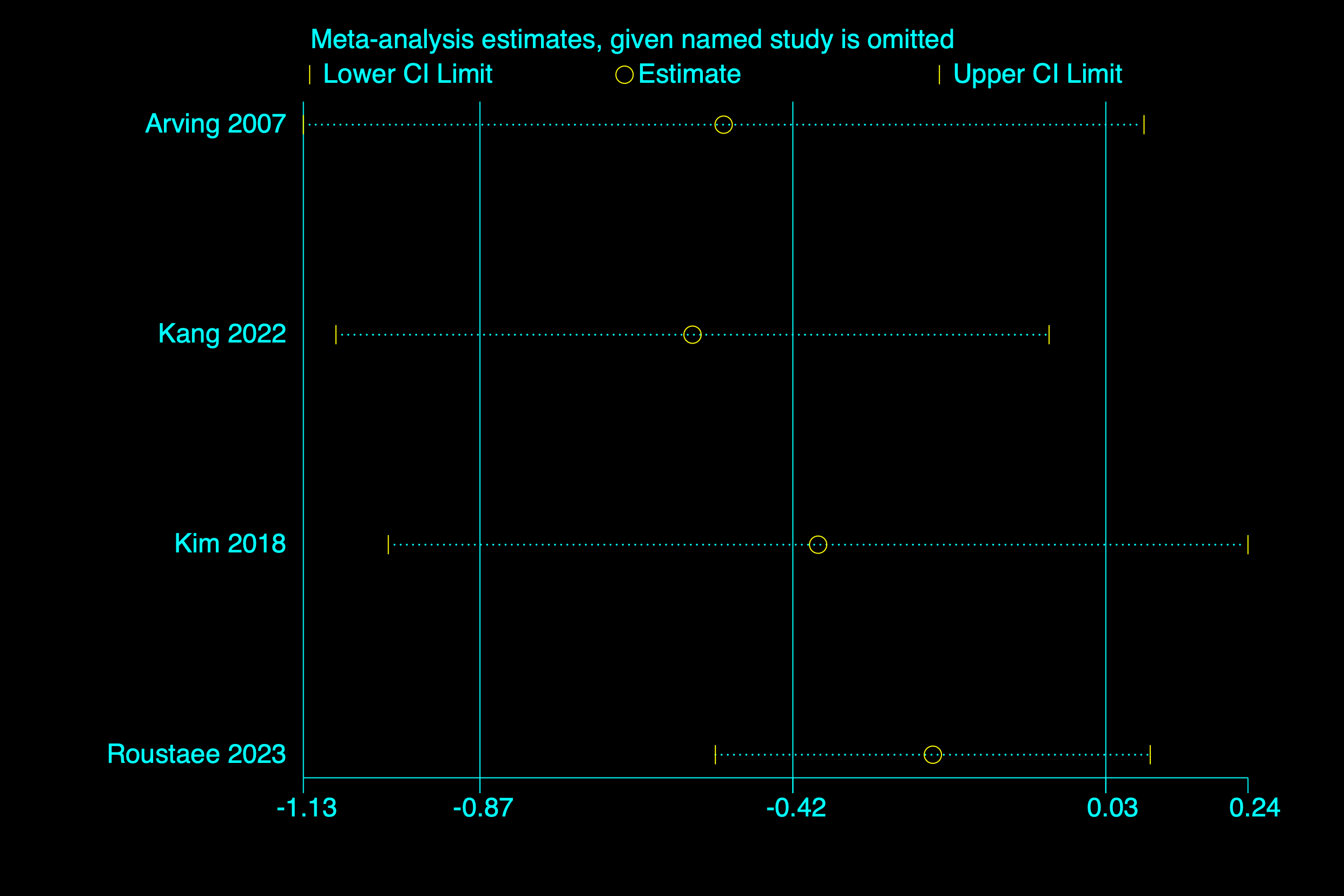

Supplement: Supplementary file 10 — Supplementary Material 10: Supplementary Fig.9: Sensitivity analysis plot for pain. Footnote: Open circles represent the pooled standardized mean difference (SMD) after omitting the named study. Horizontal dotted lines show the corresponding 95% confidence intervals. The central vertical solid line indicates the overall pooled SMD including all studies, and outer vertical lines indicate the 95% confidence interval of this overall estimate. [file 12912_2026_4505_MOESM10_ESM.jpg]

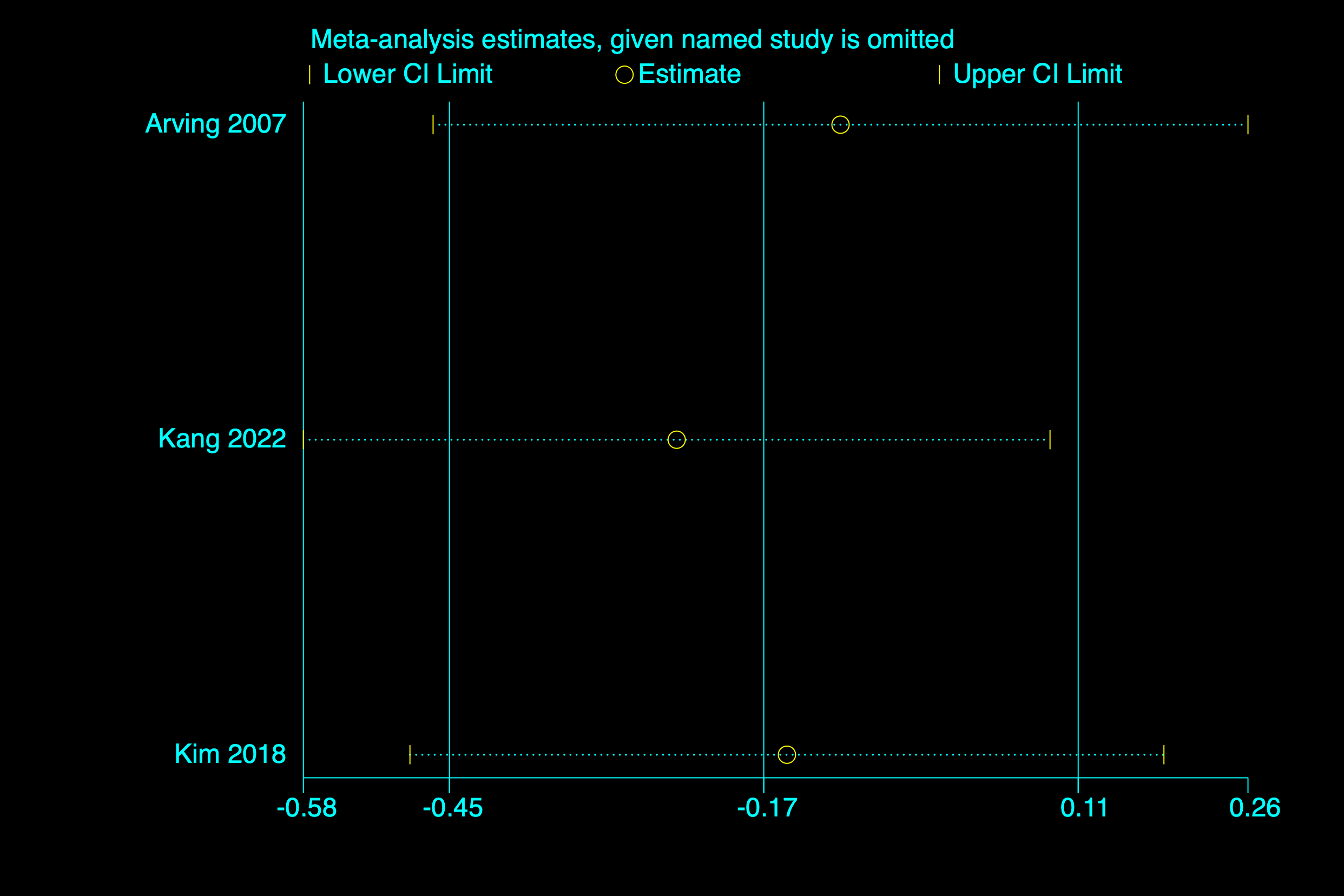

Supplement: Supplementary file 11 — Supplementary Material 11: Supplementary Fig.10: Sensitivity analysis plot for dyspnea. Footnote: Open circles represent the pooled standardized mean difference (SMD) after omitting the named study. Horizontal dotted lines show the corresponding 95% confidence intervals. The central vertical solid line indicates the overall pooled SMD including all studies, and outer vertical lines indicate the 95% confidence interval of this overall estimate. [file 12912_2026_4505_MOESM11_ESM.jpg]

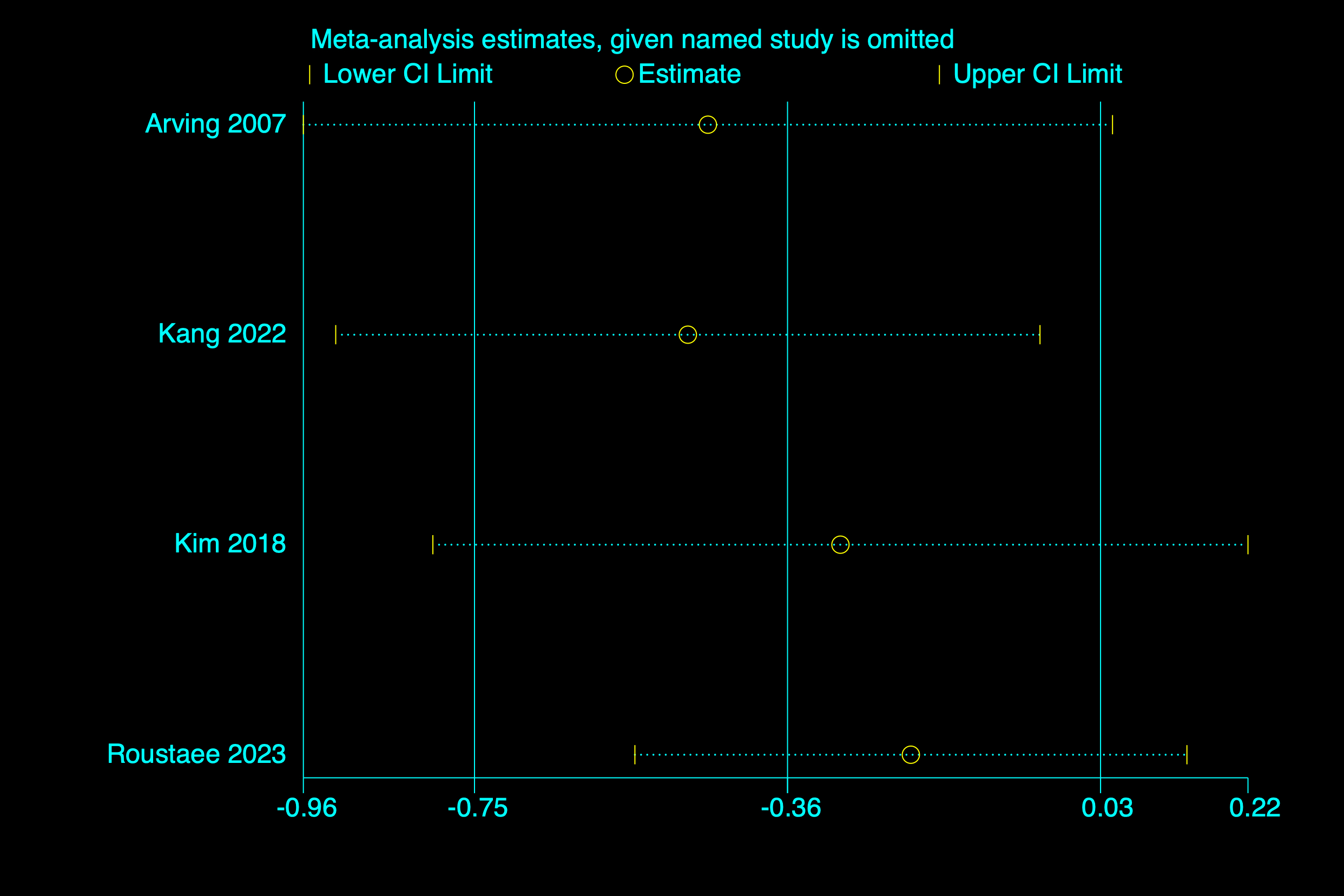

Supplement: Supplementary file 12 — Supplementary Material 12: Supplementary Fig.11: Sensitivity analysis plot for nausea/vomiting. Footnote: Open circles represent the pooled standardized mean difference (SMD) after omitting the named study. Horizontal dotted lines show the corresponding 95% confidence intervals. The central vertical solid line indicates the overall pooled SMD including all studies, and outer vertical lines indicate the 95% confidence interval of this overall estimate. [file 12912_2026_4505_MOESM12_ESM.jpg]
